# Quantitative live-cell imaging of auxin and cytokinin signalling in developing feeding sites of *Heterodera schachtii*

**DOI:** 10.1080/15592324.2026.2629039

**Published:** 2026-02-23

**Authors:** Matthijs Oosterbeek, Pepijn Bais, Hein Overmars, Jan Willem Borst, Jaap Bakker, Aska Goverse

**Affiliations:** a Laboratory of Nematology, Department of Plant Sciences, Wageningen University, Wageningen, The Netherlands; b Plant Cell and Developmental Biology, Division of Biological and Environmental Sciences and Engineering, King Abdullah University of Science and Technology, Thuwal, Kingdom of Saudi Arabia; c Laboratory of Biochemistry, Department of Biomolecular Sciences, Wageningen University, Wageningen, The Netherlands

**Keywords:** Auxin, confocal microscopy, cyst nematodes, cytokinin, feeding cell, live-cell imaging, nematode parasitism

## Abstract

Cyst nematodes are sedentary endoparasites that establish unique feeding structures (syncytia) inside their host roots. Crosstalk between auxin and cytokinin signaling is thought to play a crucial role in syncytia formation. However, the relative differences in space and time between auxin and cytokinin signaling during infection are largely unknown. Here, we used an auxin‒cytokinin double-reporter line to study the spatio-temporal dynamics of auxin and cytokinin signaling during the initiation and expansion of syncytia in *Arabidopsis thaliana* roots induced by the beet cyst nematode *Heterodera schachtii*. Continuous live-cell imaging of single juveniles showed that upon infection, cytokinin signaling increases rapidly, precedes auxin signaling, and occurs in nearly all cell types, also outside the syncytial area. Auxin signaling is restricted to the syncytial area, starts after a lag phase, and is induced after the first contours of the syncytial area have become visible. Within the syncytial area, the signaling domains overlap, and the elevated auxin response in nuclei is associated with a high cytokinin response. Quantitative analyses showed that cytokinin signaling is relatively high in cyst nematode infection sites when compared to signaling maxima in root tips, while auxin signaling is relatively weak when compared to signaling maxima in root tips.

## Introduction

Plant‒parasitic nematodes cause an estimated annual crop yield loss between 8.8% and 14.6% worldwide, equaling 157 billion dollars.^1^^,^^2^ Among these plant‒parasitic nematodes, the root-knot nematodes of the genus *Meloidogyne* and the cyst nematodes of the genus *Heterodera* and G*lobodera* are among the top 10 most harmful nematodes.^3^ Owing to this, the best-studied species of plant‒parasitic nematodes are among the root-knot nematodes and cyst nematodes. These sedentary nematodes can feed on monocot and dicot plants, establishing a unique feeding structure inside the plant root through a complex interaction at the cellular and molecular level.^4^ Cyst and root-knot nematodes hatch from their eggs, and second-stage juveniles search for a host to invade the roots using distinct modes of action. Cyst nematodes move intracellularly through the plant's cortex, where they select an initial feeding cell in or near the vascular bundle.^5^ Subsequently, they inject this cell with a suite of secretory compounds known as effectors that start to alter the host's metabolism, leading to the formation of a syncytium.^6^ This syncytium is formed through the partial breakdown of neighboring cell walls and followed by fusion of their cell membranes.^7^ Second-stage juveniles of root-knot nematodes, however, enter the root near the meristematic zone and move intercellularly through the vascular bundle. There, they select up to 10 individual cells and transform them into giant cells by injecting effector compounds that induce cell division without cytokinesis.^8^ Although cyst and root-knot nematodes develop distinct feeding structures, they share common characteristics, as both form large multinucleate cells with a proliferated endoplasmic reticulum through reprogramming of normal root cells.^9^ Feeding site formation, in general, is paired with aberrant cell cycle activation and drastic changes in gene transcription.^10^ Feeding sites deplete the host of resources and function as the sole nutrient source for the parasite. Interruption or prevention of the formation of these feeding sites leads to the demise of the nematode.^11^

The infection process by cyst and root-knot nematodes is irrevocably linked to changes in the hormone balance of the plant. Together with cytokinins, auxins are the key plant hormones involved in cell division and differentiation and also play a significant role in feeding site initiation and development. The phytohormone auxin, indole-3-acetic acid (IAA), is considered to play a pivotal role in the feeding site formation of both cyst- and root-knot nematodes and is thought to be responsible for many of these processes, such as hypertrophy, cell wall ingrowths, and cell cycle activation.^12^ Several auxin mutants in *Arabidopsis thaliana*, such as *dgt*, *aux1*, and *arf7/arf19,* are significantly less susceptible to either root-knot or cyst nematode infection.^13-15^ Moreover, the auxin-responsive DR5 reporter revealed that cyst nematode-induced feeding site formation is accompanied by a local and transient auxin accumulation as early as 18 h post infection.^13^^,^^16^ IAA accumulation seems to occur in expanding syncytia and in cells surrounding the syncytia where the majority of these neighboring cells will be incorporated into the syncytium at a later stage of development. In addition, auxin is suspected to trigger distinct developmental processes during cyst and root-knot nematode infection. For example, auxin-induced LATERAL ORGAN BOUNDARIES-DOMAIN (LBD) 16 expression is important for the formation of secondary roots and is upregulated in giant cells of root-knot nematodes but it is downregulated during cyst nematode infection.^17^^,^^18^ Reporter and transcriptome studies have investigated the expression of auxin-related genes at various days after infection of root-knot nematodes, and these snapshots suggest a dynamic and complex spatial as well as temporal expression pattern of auxin-related genes, such as the PINs and GRETCHEN HAGEN 3 (GH3).^14^^,^^19^ Similarly, reporter studies have shown the involvement of auxin-related genes in the infection process of cyst nematodes, such as ARF and PIN, with an equal complex expression pattern.^20^^,^^21^ In these studies, auxin biosynthesis and auxin-response genes, such as tryptophan decarboxylase (TDC) and (GH3), are predominantly up-regulated at the early stages of nematode infection, where in contrast auxin repressors like the IAA family, are downregulated. From this, it can be inferred that the accumulation itself likely arises, at least partially, through changes in local auxin biosynthesis and catabolism as well as auxin transport.^22^

The phytohormone cytokinin, similar to auxin, is known to play an important role in nematode feeding site development. In plants, cytokinins exert cell cycle control, are able to delay senescence and convert tissues into sinks by modulating nutrient translocation.^23^ Owing to these properties, they have long been thought to play a role during nematode infection. Several cytokinin-insensitive mutants are less susceptible to cyst or root-knot nematode infection like *ahp1/2/3*, *ahk4*, and *arr1/12* as well as those with reduced cytokinin levels, such as *CKX3* and *CKX4*.^24-27^ Cytokinin has been shown to accumulate in the feeding site during the infection cycle of both cyst and root-knot nematodes using the cytokinin reporters TCSn:GFP and ARR5:GUS.^24^^,^^26^ These studies show that cytokinin expression occurs in the same timeframe compared to auxin but is induced later, from at least 4 dpi to 14 dpi.^24^^,^^25^^,^^27^ Moreover, cytokinin reporter signals are also present in the surrounding tissue of both cyst and root-knot nematode-feeding cells. In addition, they can be found in the syncytium of cyst nematodes but are curiously not inside the giant cells of root-knot nematodes.^28^ These differences are also observed in transcriptomic studies for the expression of cytokinin biosynthesis, catabolism, and signaling genes, which is attributed to the underlying difference in cell cycle progression during syncytium and giant cell formation.^24^ This was demonstrated by the importance of the cytokinin signaling gene *AhK4* in syncytium development, while *AhK2* and *AhK3* were shown to be crucial for giant cells development.^24^^,^^27^

Although auxin and cytokinin signaling have been studied separately, it can be inferred from previous studies that auxin and cytokinin responses occur simultaneously in feeding sites of both cyst and root-knot nematodes. This is remarkable, given the antagonistic nature of auxin and cytokinin signaling in normal plant root development.^29^ For example, auxin decreases the expression of the cytokinin biosynthesis gene CYP735A in *Arabidopsis* but also directly activates two type A ARRs, ARR7 and ARR15, thereby inhibiting cytokinin signaling.^30^^,^^31^ Conversely, cytokinin modulates auxin metabolism and transport by, for example, affecting IAA17, which is important in maintaining auxin levels and regulating the expression of the PIN auxin export family.^32^^,^^33^ However, auxin and cytokinin interactions are not always antagonistic, as additive and synergistic effects also occur, like auxin-mediated regulation of cytokinin biosynthesis and their collaborative role in cell divisions in vascular tissues. In this case, the cytokinin biosynthesis genes IPT5 and IPT7 are upregulated in *Arabidopsis* roots in response to auxin treatment through *SHY2/IAA3* expression.^34^^,^^35^ In addition, auxin decreases the expression of the cytokinin catabolism genes CKX2, CKX4, CKX7, and allows cytokinin to promote vascular differentiation and regeneration.^36^^,^^37^ Interestingly, vascularization is also associated with cyst and root-knot nematode feeding site formation and may be the result of auxin‒cytokinin cross talk.^38^^,^^39^ However, current auxin and cytokinin data are based on independent studies, and it is *de facto* unknown if and how auxin and cytokinin signaling events merge during feeding site initiation and development. Moreover, as they have never been investigated simultaneously in this context, it is also unknown whether auxin and cytokinin signaling occurs simultaneously or consecutively within feeding sites. Additionally, previous analytical approaches have favored qualitative assessments over quantifiable numerical data by evaluating results as either increased, elevated or high but not quantified.^13^^,^^24^^,^^26^ The spatial and temporal dynamics are further obfuscated by examining non-synchronous infection sites based on daily observations, thereby observing only general trends.

Therefore, the aim of this study was to monitor the spatio-temporal dynamics of auxin and cytokinin signaling simultaneously in individual feeding sites of the cyst nematode *H*. *schachtii* using quantitative live-cell confocal imaging. For this, roots of an *Arabidopsis* reporter line were infected *in vitro* containing a double-reporter construct consisting of the auxin-responsive DR5revV2 promoter driving the expression of a gene encoding a nuclear targeted green fluorescent protein (n3GFP) and the cytokinin responsive promoter TCSn driving nuclear targeted ntdTomato gene expression. Upon infection, single juveniles were observed and selected when they were in the sedentary phase 18 h after inoculation. Next, they were transferred to a mini-growth chamber for continuous growth and time lapse recording of dual reporter gene expression for up to 140 h using confocal laser scanning microscopy (CLSM). Every 30 min, 7–10 optical slices were collected, capturing the induction and development of a syncytium, including changes in its surrounding root tissues. In total, a collection of more than 6000 optical slices was obtained to monitor the spatio-temporal dynamics of auxin and cytokinin signaling during feeding site formation. Quantitative and comparative analyses of these data revealed that the signaling dynamics of auxin and cytokinin show distinct patterns in time and space. Moreover, we show that simultaneous monitoring of auxin and cytokinin signaling using live-cell imaging not only uncovers overlap of auxin and cytokinin signaling domains within the syncytial area but also reveals that elevated auxin signaling is associated with high cytokinin responses in the same cells during feeding site formation. In addition, our data suggest that the initial phase of syncytia development may be independent of auxin signaling. The possible implications of cross-talk between auxin and cytokinin as well as their independent roles in feeding site formation by cyst nematodes are discussed.

## Materials and methods

### Plant material

Seeds containing a DR5revV2:n3GFP-nls and TCSn:ntdTomato-nls transcriptional fusion are available in a Col-0 background where the auxin‐responsive promoter DR5^40^ drives the expression of n3GFP and the cytokinin-responsive promoter TCSn^41^ the expression of ERFP. These seeds were kindly obtained from Prof. Dr. Dolf Weijers, Wageningen University and Research, The Netherlands. Both fluorophores are expressed with a nuclear localization signal (NLS) to concentrate the fluorescent signal in the nucleus for enhanced sensitivity and accurate quantification irrespective of cell size or vacuolization status.^42^ The seeds were stratified at 4 °C for 4 d and subsequently vapor sterilized (50 ml H_2_O, 40 ml NaOCl 5%, and 4.4 ml HCl 25%) for a duration of 3.5 h. Seeds were plated on solid KNOP medium (KNOP minerals, 10% sucrose, 0.8% Daichin agar, and pH 6.4).^43^ The plates were incubated horizontally at 21 °C under a 16 h light/8 h dark cycle for 3 d to ensure the growth of the root into the agar medium. The plants were subsequently placed vertically to promote the growth of the roots along the bottom.

### Nematode hatching and sterilization

*Heterodera schachtii* cysts were harvested from the soil and cleaned using 1% NaN_3_ for 20 min. The cysts were subsequently incubated on a 100 μM sieve at room temperature in the dark for 4 d in a 3 mM ZnCl_2_ solution to promote the hatching of infective juveniles. To prevent contamination, the antibiotics 0.15% gentamycin (w/v), and 0.05% nystatin (w/v) were added to the solution. The freshly hatched second-stage juveniles (J2) were subsequently sterilized as described previously.^13^ They were collected through purification using a sucrose (35%) gradient. The infective juveniles were surface sterilized (0.002% Triton X-100 v/v, 0.004% NaN_3_ w/v, and 0.004% HgCl_2_ w/v) for 10 min and afterwards rinsed 3× in sterile tap water. Next, they were suspended in 0.7% Gelrite (Duchefa Biochemie) solution to a final concentration of 10 juveniles/μl for use as inoculum in nematode infection assays.

### *In vitro* nematode infection assay

**Figure 1.**
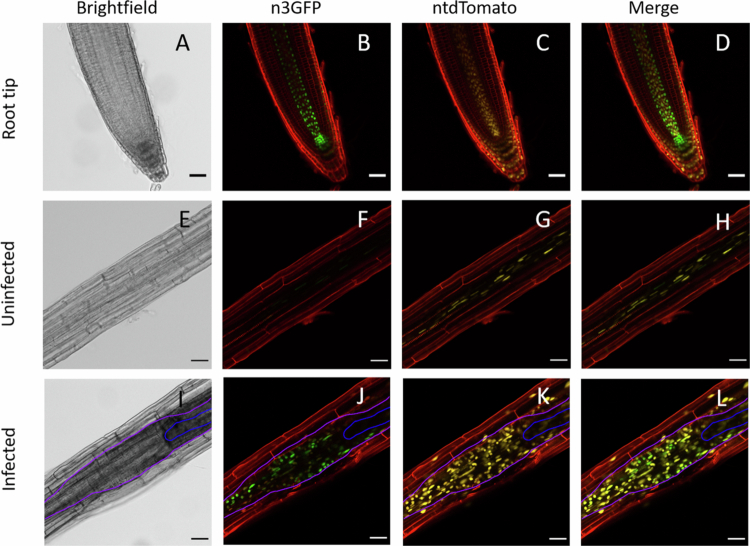
Visualization of cytokinin and auxin signaling through the use of the double-reporter DR5revV2:n3GFP-TCSn:ntdTomato in uninfected roots and *Arabidopsis* roots infected with *H. schachtii* at 4 DPI. (A–D) Auxin (green) and cytokinin (yellow) signaling in uninfected *Arabidopsis* root tip. The scale bar represents 20 μM. (E–H) Auxin and cytokinin signaling in the mature zone of uninfected *Arabidopsis* root. The scale bar represents 40 μM. (I–L) Root infected with *H. schachtii* at 4 DPI. The blue line shows the contour of the nematode, and the pink line outlines the syncytial area. The scale bar represents 40 μM. The cell walls are stained with propidium iodide (red). The data displayed is a representative image of over 25 observations from three independent replicates with similar results using a confocal laser scanning microscope (CLSM).

For functional validation of the auxin‒cytokinin double-reporter DR5revV2:n3GFP-nls/TCSn:ntdTomato-nls (Figure 1), both infected and uninfected root systems were imaged using the standard method to monitor the spatio-temporal distribution of fluorescent reporter genes in cyst nematode-induced feeding sites.^26^ One-week-old seedlings were inoculated with 50 juveniles and imaged at 0-, 1-, 2-, 3-, 4-, and 7-d post-inoculation. For each time point ≥25 feeding sites were selected from a set of 10 plants. The samples were stained with 100 μM propidium iodide for 10 min before being imaged under an inverted LEICA SP8 confocal microscope. For detection of auxin signaling, the n3GFP fluorophore was excited with a 488 nm argon laser, and emission was captured at 505–530 nm. Cytokinin signaling was detected upon excitation of ntdTomato with a 543 nm argon laser, and emission was captured at 560–580 nm. Propidium iodide was excited at 488 nm, and emission was captured at >650 nm to visualize the cell boundaries.

### *In vivo* live-cell imaging using confocal laser scanning microscopy (CLSM)

**Figure 2.**
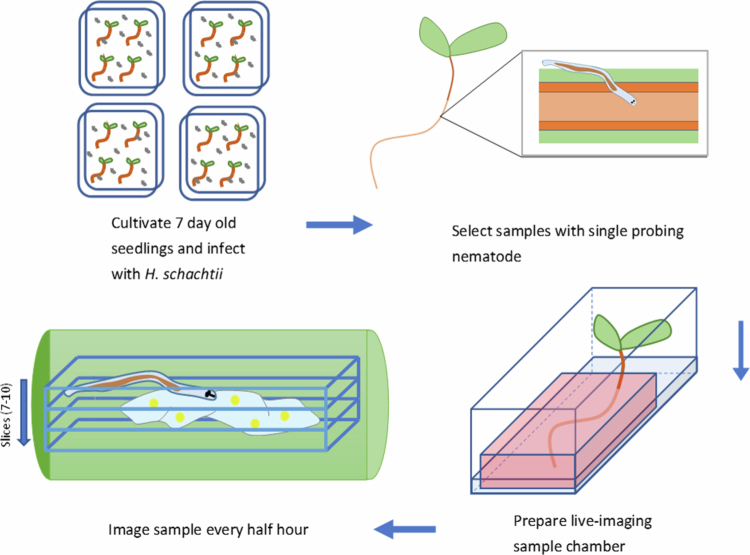
Schematic outline of all steps involved in the selection of sedentary second-stage juveniles from *H. schachtii* for live-cell imaging using a mini-chamber set up as described previously (see Materials and methods). Seven-day-old infected samples were observed, and those with a single nematode at the onset of feeding site initiation were selected. Three to five samples are prepared in a live imaging chamber, and one is imaged. Using optical sectioning with CLSM, a major part of the syncytium can be captured using ~7 optical slices (z-stack).

Plant roots with a single nematode infection were used for *in vivo* live-cell imaging. Around 200 DR5v4:EGFP-nls/TCSn:ERFP-nls seeds were germinated on square plates containing KNOP and inoculated with approximately 20 juveniles per plant six days later, as described above. After 19 h of incubation at 21 °C under a 16 h light/8 h dark cycle, the plants were screened using a binocular for nematode juveniles that successfully invaded the roots and had transitioned from their migratory phase. During cyst nematode migration, the nematode uses its stylet to penetrate the plant's cell walls and move through them. Near the vascular bundle, the nematode stops migrating and enters its sedentary phase, where it selects an initial syncytial cell. It probes this cell using its stylet before penetrating it and starting a cycle of feeding from the cytoplasm and injecting effector compounds.^44^ To monitor nematodes from the start of their sedentary period, samples were collected with nematodes that were visibly probing root cells and selecting an initial syncytial cell. Up to five of these samples were taken, and their roots were stained with propidium iodide for 10 min. These seedlings were placed into sterile chambered coverglasses (Nunc™ Lab-Tek™ Chambered Coverglass) in a flow cabinet (Figure 2). The chambers enable the seedlings to grow in an enclosed sterile environment, and the thin coverslip bottom facilitates imaging through an inverted CLSM. Subsequently, a slice of sterile solid KNOP medium supplemented with 10 μM propidium iodide was carefully placed on top of the root, leaving the shoot free, to bring it close to the imaging surface and minimize sample drift. Afterwards, the chambers were filled with 2 ml of liquid KNOP medium supplemented with 10 μM propidium iodide to maintain constant propidium iodide concentrations. Finally, the chambers were sealed with parafilm to secure a sterile environment during imaging, which still allows for gas exchange.

The prepared samples were imaged on a ZEISS LSM510/CONFOCOR2 confocal microscope equipped with a 10× air objective (NA 0.4) and an argon ion laser. The argon laser was used for excitation of GFP at 488 nm, and a He/Ne laser was used to excite ntdTomato at 543 nm. GFP fluorescence emission was detected with a bandpass filter at 505–550 nm, while ntdTomato fluorescence was detected with a 560–600 bandpass filter. The optical slices were acquired in confocal mode (1 Airy unit) with an average of 8 scans. At 19 h post infection, the samples were monitored for 160 h, and z-stack images were taken every half-hour. The reporter used for auxin and cytokinin signaling was excited and measured in the same manner as described above during the nematode infection assay. All time series are available at the Supplementary data.

### Testing sample viability

Before live-cell imaging was performed, it was confirmed that the infected seedling in the sample chamber was viable. First, it was investigated what the effect of different cell-stains was on the growth of the seedlings. *Arabidopsis* seedlings were allowed to germinate and grow for 7 d on KNOP medium in square plates as described above. The medium was supplemented with various concentrations of Calcofluor White or propidium iodide to determine the effect of these cell stains on the growth of the plant. For Calcofluor White, concentrations of 0%, 0.1%, 1%, and 10% (w/v) were used, and for propidium iodide, concentrations of 0 μM, 10 μM, 50 μM, and 100 μM were used. The samples were incubated vertically at 21 °C under a 16 h light/8 h dark cycle for 7 d. In addition, infected samples in chambered coverglass were prepared as described above and stored in a climate chamber (Nunc™ Lab-Tek™ Chambered Coverglass) to assess the viability of the nematode in this setup. After three weeks, the samples were visually inspected for the presence of J3 and J4 females.

### Data analysis

Image analyses were carried out using Fiji (v2.0.0-rc-69).^45^ CLSM allows for imaging optical slices of the sample, and a stack of these optical slices is known as a z-stack. Z-stacks were taken every 30 min during live-cell imaging, showing the fluorescence in the nuclei of infected and control root tissue as the result of promoter activity. Z-stacks consisted of ~7 slices of 67 µm and included tissue above and below the nematode.

**Figure 3.**
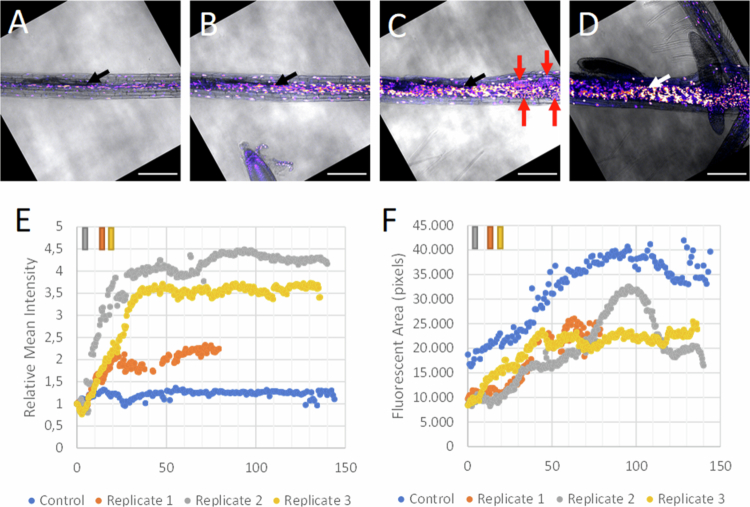
Cytokinin signaling revealed by the double-reporter DR5revV2:n3GFP-TCSn:ntdTomato with continuous live-cell imaging in *Arabidopsis* roots infected with *H. schachtii*. Snapshots from the movie of the 3rd replicate (Rep3) at (A) the start of live-cell imaging (*t* = 0), (B) at the first appearance of the syncytial area (*t* = 21.5 h), (C) during expansion of the syncytial area (*t* = 68 h) and (D) at the end of the measurements with fourth-stage juveniles and outgrowing secondary roots (*t* = 130 h). The black and white arrows indicate the head of the nematode, and the red arrows show the developing root primordia. The scale bar represents 200 μm. The fluorescence intensity is displayed as a blue < orange < white (fire) gradient. (E) The average intensity per pixel of fluorescent cytokinin signaling nuclei in one optical slice per timepoint relative to the first measurement (*t* = 0) (RMI) of the entire imaged primary root for each replicate. The bars on the upper left indicate the time at which the syncytial area was first observed with bright-field microscopy. (F) The number of fluorescent pixels (FA) in the main root for each replicate in one optical slice per timepoint. The root tip is located to the left of the image.

**Figure 4.**
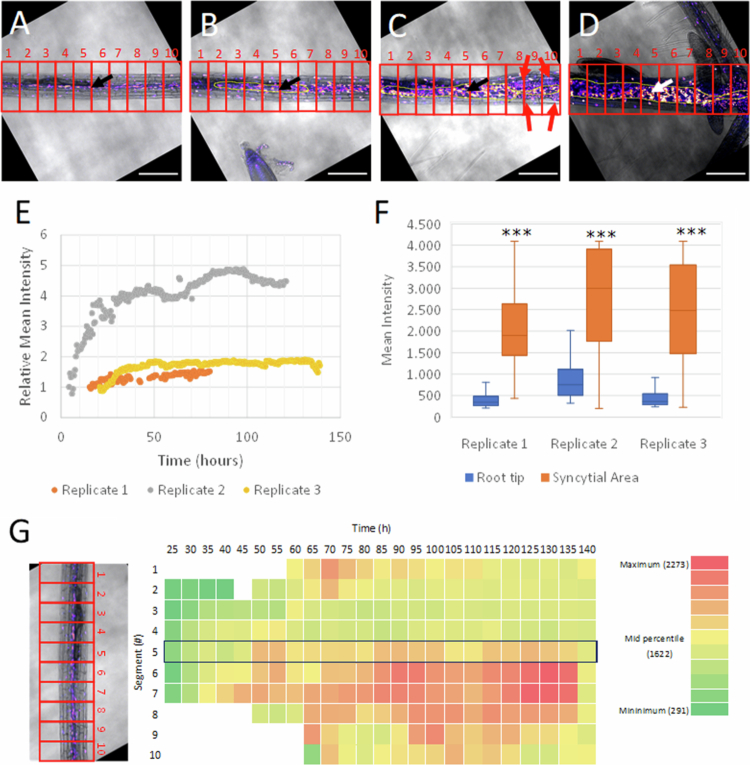
Analysis of cytokinin signaling dynamics within the syncytial area induced by *H. schachtii* in a root of *Arabidopsis* revealed by the double-reporter DR5revV2:n3GFP-TCSn:ntdTomato with continuous live-cell imaging. Snapshots from the movie of the 3rd replicate (Rep3) at (A) the start of live-cell imaging (*t* = 0), (B) at the first appearance of the syncytial area (*t* = 21.5 h), (C) during expansion of the syncytial area (*t* = 68 h), and (D) at the end of the measurement (*t* = 130 h) showing the emergence of a fourth-stage juvenile and secondary roots. The border of the syncytial area is indicated in yellow, and the root is divided into 10 optical segments indicated in red. The black and white arrows indicate the head of the nematode, and the red arrows represent the developing root primordia. The fluorescence intensity is displayed as a blue < orange < white (fire) gradient. The scale bar represents 200 μm. (E) Relative mean intensity of the TCSn:ntdTomato signal (RMI_tomato_) measured inside the marked syncytial area for all three biological replicates. (F) Mean intensity of cytokinin signaling in nuclei in the syncytial area compared to nuclei in the root tips of secondary roots (*t* = 71.5 h (Rep1); 97.5 h (Rep2); and 130.5 h (Rep3)). Significance was determined using a two-tailed homoscedastic *t-*test. ***p < 0.001 (G) Heatmap of the average fluorescence intensity within the syncytial area per root segment over time for the 3rd replicate (Rep3). Red indicates a higher intensity, and the optical segments are 90 μm long. The segment with the head of the nematode (#5) is marked by a blue border. The root tip is located to the left of the image.

To monitor temporal changes in auxin and cytokinin signaling throughout the infection area, for each timepoint, the optical slice with the head of the nematode was analyzed for fluorescence intensity derived from n3GFP or ntdTomato expression by calculating the mean fluorescence intensity (MI) per optical slice in the main root. This intensity was only measured for pixels selected through the use of a mask to select for nuclei. This mask was created for pixels above the noise threshold using the ‘default’ ImageJ thresholding value. The artifacts in the mask were smoothed out with the ‘smooth’ and ‘despeckle’ function and contrast was enhanced with the normalized ‘enhance contrast’ function. Jagged edges were corrected with the ‘dilate’ and ‘erode’ functions. The mean intensity (MI) of each timepoint was then normalized by division with the mean intensity of the first measurement in the time series (*t* = 0) (RMI). In addition to the relative mean intensity (RMI), the fluorescent area (FA) was defined as the sum of all fluorescent pixels as detected in fluorescent nuclei at a given timepoint for the observed optical slice. This RMI and fluorescence area (FA) were plotted in a graph to show the overall changes in the fluorescence of the nematode-infected root areas over time as a result of reporter gene activity (Figures 3E and F, 4E and F). This was done for three independent biological replicates.

**Figure 5.**
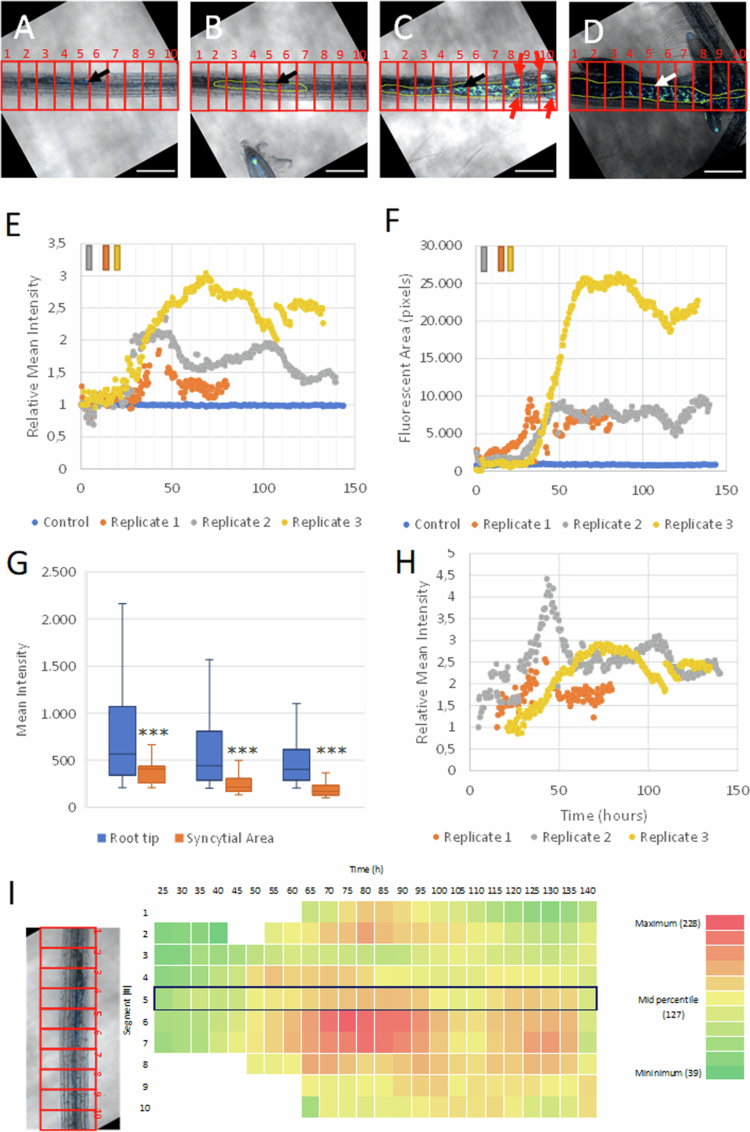
Analysis of auxin signaling dynamics within the syncytial area induced by *H. schachtii* revealed by the double-reporter DR5revV2:n3GFP-TCSn:ntdTomato with continuous live-cell imaging. Snapshots from the movie of the 3rd replicate (Rep3) at (A) the start of live-cell imaging (*t* = 0), (B) at the first appearance of the syncytial area (*t* = 21.5 h), (C) during expansion of the syncytial area (*t* = 68 h), and (D) at the end of the measurement (*t* = 130 h), showing the emergence of fourth-stage juvenile and secondary roots. The border of the syncytial area is indicated in yellow, and the root is divided into 10 optical segments indicated in red. The black and white arrows indicate the head of the nematode, and the red arrows represent the developing root primordia. The fluorescence intensity is displayed as a blue < green < white (green fire blue) gradient. The scale bar represents 200 μM. (E) The average intensity per pixel of fluorescent auxin signaling nuclei in one optical slice per timepoint relative to the first measurement (*t* = 0) (RMI) of the entire imaged primary root for each replicate. The bars on the upper left indicate the time at which the syncytial area was first observed with bright-field microscopy. (F) The number of fluorescent pixels (FA) in the main root for each replicate in one optical slice per timepoint. The bars on the upper left indicate the timepoint at which the syncytial area was first observed. (G) Mean intensity of auxin signaling in the nuclei of the syncytial area compared to nuclei in secondary root tips (*t* = 42.5 h (Rep 1); 46.5 h (Rep2) and 76.5 h (Rep3)). Significance was determined using a two-tailed homoscedastic *t-*test. ***p < 0.001 (H) relative mean intensity (RMI) of the DR5revV2:n3GFP signal measured inside the marked syncytial area for all three biological replicates. (I) Heatmap of the average fluorescence intensity within the syncytial area per optical root segment (90 μM) over time for the 3rd replicate. Red indicates a higher fluorescence intensity, and the segment that includes the head of the nematode is marked by a blue border. The root tip is located to the left of the image.

To determine the temporal changes/dynamics in fluorescence intensities more specifically in the nematode feeding sites, a single optical slice was selected from the z-stack for every timepoint. The optical slice was chosen so that the image always contained the head of the nematode. Because small aberrations in the sample position might shift specific root segments to different optical slices, using the head of the nematode as a reference allows the measurement to be performed at the same depth in the root even if this depth is not always the same in the z-stack. The feeding site was subsequently outlined manually (region of interest (ROI) selection) for various timepoints in a series by merging the brightfield and fluorescence images per optical slice and using that as a reference. In this way, the induction and development of the feeding cell in the selected nematode-infected root area was confirmed. Between these manually outlined feeding sites at various time points, the development of the syncytium was interpolated thereby simulating syncytium growth (ROI interpolation). The mean fluorescence intensity (MI) of the reporter was then measured inside these feeding cell-enriched root tissues using the previously described mask. These mean intensities were plotted relative to the first measurement (RMI) in a graph to show the overall changes in fluorescence of feeding cell-enriched root areas over time as the result of reporter gene activity relative to the first measurement (Figures 4E and 5H). This was done for three independent replicates.

For the comparison of the intensity of auxin or cytokinin signaling in the syncytial area with the signaling intensities in secondary root tips, a similar approach as above was used. Using the above-described mask, the intensities were collected within the outlined syncytium at one timepoint. This timepoint with the highest signaling intensity in the sample was selected based on the previous analysis (Figures 3E and F and Figure 4E and F). Similarly, the area of the secondary root tip with the highest signaling intensity was selected for the root tips that were in focus and closest in time to the timepoint in which the syncytium was measured (Supplementary Figure S9). In this area, the (pixel) intensities were collected and displayed in a box graph (Figures 4F and 5G).

To determine how auxin and cytokinin signaling changes outside the syncytial area, a segment analysis was performed. For these spatial segment measurements, again a single slice time series containing the head of the nematode was used. In every slice, the length of the root is then marked in a rectangular area (ROI selection), and with that area, the syncytial area was marked as above and filtered through the mask. For this selection, the mean intensity (MI) was measured for every column of 90 μm within the rectangle. The mean signal intensity (MI) for that column is then averaged across 10 measurements over time (5 h) of that column. This gives the average signal intensity for that particular region at that particular depth over that time period (Figures 4G and 5I).

**Figure 6.**
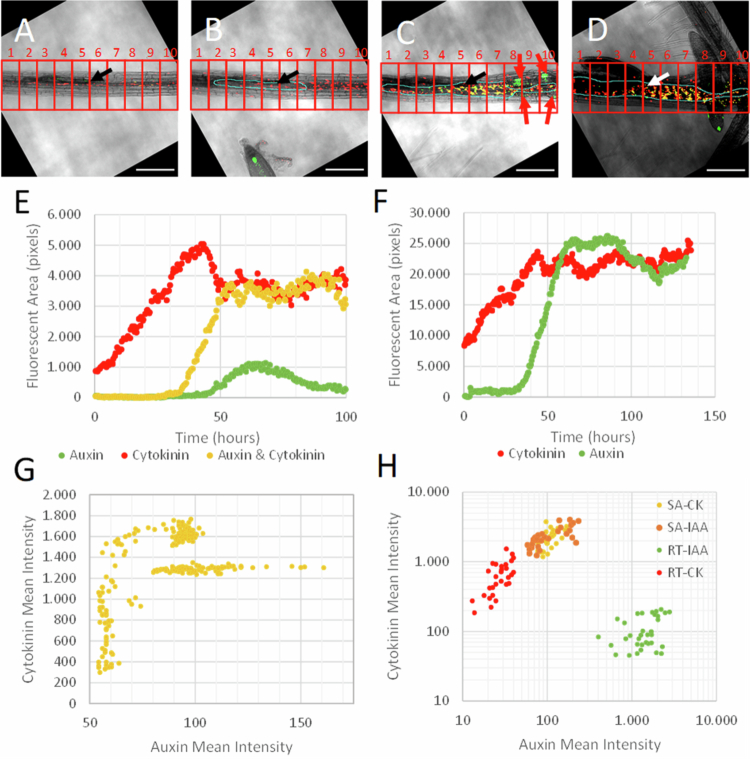
Comparative analyses of auxin and cytokinin signaling dynamics during the development of syncytia induced by *H. schachtii* using the *Arabidopsis* DR5revV2:n3GFP-TCSn:ntdTomato double-reporter line. Auxin (green) and cytokinin (red) signals were merged, resulting in a yellow signal when they overlap in the syncytial area of a single replicate (Rep3) at (A) the start of live-cell imaging (*t* = 0), (B) at the first appearance of the syncytial area (*t* = 21.5 h), (C) during expansion of the syncytial area (*t* = 68 h), and (D) at the end of the measurement (*t* = 130 h), showing the emergence of a fourth-stage juvenile and secondary roots. The black and white arrows indicate the head of the nematode, and the red arrows show the developing root primordia. The scale bar represents 200 μm. (E) The fluorescent area (FA) as an indication of the number of nuclei that show auxin signaling, cytokinin signaling or both, as measured over time in the entire image of the main root infected with *H. schachtii* (Rep3) using a binary masking method. (F) Fluorescent area (FA) of auxin and cytokinin signaling in main root infected with *H. schachtii* (Rep3) showing a delay for auxin signaling. (G) The mean intensity of the cytokinin signal plotted against the mean intensity of the auxin signal in the entire main root for all measured time points of the 3rd replicate (Rep3), showing the auxin and cytokinin ratios measured at intervals of 30 min during a period of 140 h (H) The mean intensity of auxin and cytokinin signals found at a single time point (*t* = 113 h) in the 3rd replicate in single nuclei. At least thirty nuclei were selected from the top 10% highest auxin and cytokinin signaling nuclei located in the syncytial area (NFS-IAA and NFS-CK), as well as nuclei present in secondary root tips (RT-IAA and RT-CK), resulting in three groups of cell types based on their auxin‒cytokinin ratios. For further explanation, see the main text. The root tip is located to the left of the image. Abbreviations: IAA—indole-3-acetic acid, CK—cytokinin, SA—syncytial area, and RT—root tip.

In order to determine which nuclei displayed auxin signaling, cytokinin signaling or both, a binary masking method was used instead of a masking method for nuclei. Using the same single-slice time series as mentioned above, all auxin and cytokinin signals above the noise threshold (using the ‘default’ ImageJ thresholding value) were maximized, while all other signals were removed to create the binary mask. The maximized auxin signal then overlapped with the maximized cytokinin signal, and pixels were colored based on whether the maximized auxin signal, maximized cytokinin signal, or both were present. This image was then mapped back onto the corresponding brightfield images to display where overlap occurred (Figure 6A–D). The number of pixels for each signaling type was subsequently counted and displayed in a graph (Figure 6E).

To display the changes in the fluorescent area over time, the auxin and cytokinin signals were (Figure 6E) to display the delay in the increase in auxin signaling (Figure 6F). The correlation between auxin and cytokinin signaling intensity data as seen in Figure 6G is created by plotting the mean intensity of cytokinin signaling in the main root (Figure 3E) against the mean intensity of auxin signaling in the main root (Figure 4E) for all measured time points of the 3rd replicate. In Figure 6H, the auxin and cytokinin mean intensity was determined in the nuclei of the syncytial area and in the root tips. The image was thresholded to select for the top 10% of pixel intensity for both auxin and cytokinin in their respective areas. A total of 30 individual nuclei were manually selected for each group.

## Results

### A fluorescent double-reporter reveals overlapping auxin and cytokinin signaling domains in young feeding sites induced by *H. schachtii*

Prior to the establishment of a live-cell imaging system to monitor the spatio-temporal dynamics in the auxin and cytokinin signaling domains during feeding site development, a classical approach was used to investigate the expression patterns of the auxin‒cytokinin double-reporter DR5revV2:n3GFP–TCSn:ntdTomato^46^ in the roots of *Arabidopsis* seedlings infected with *H*. *schachtii* juveniles. As a reference, the roots of uninfected *Arabidopsis* seedlings were studied to validate the expression pattern of this dual-reporter system. In 11-d-old uninfected seedlings, cytokinin signaling was observed in the vascular cylinder and cortex of the mature root (Figure 1G). In a number of seedlings, a cytokinin response was also observed in the epidermis of the mature root. For the root tip, the expression of TCSn:ntdTomato has been reported for procambium, columella, epidermis, and lateral root cap cells,^46^ which is in concordance with the observed fluorescence produced by the double-reporter in our study (Figure 1C). Little to no auxin signal was observed in the root above the maturation zone (Figure 1F). The expression of DR5revV2:n3GFP was evident in the root tip (Figure 1B) and, as reported^47^, was most prominent in the quiescent center (QC) and neighboring stem cells and protoxylem and relatively low in the metaxylem, pericycle, lateral root cap, and epidermal cells. Visual inspection of the photographs (Figure 1B, C and D) reveals that high auxin and high cytokinin signaling occurs in distinct nuclei. Altogether it is concluded that the expression patterns of the auxin–cytokinin double-reporter DR5revV2:n3GFP–TCSn:ntdTomato are consistent with previous data and show that domains with high auxin and high cytokinin signaling do not overlap.

For *H. schachtii* infected root samples, a profound ntdTomato fluorescence signal was observed as compared to similar regions of uninfected roots, indicating enhanced cytokinin signaling within and around the ‘syncytial area’ at 4 d post-infection (DPI) (Figure 1K). Measurements were performed at 4 DPI as this timepoint is indicative of both the auxin and cytokinin signaling windows during early feeding site development.^21^^,^^26^ The term ‘syncytial area’ is used, since with our microscope setup, syncytial cells cannot be distinguished from neighboring cells that are not connected to the syncytium. As described in previous studies,^48^ syncytial elements are surrounded by cambial and peridermal cells. Additionally, root cells preconditioned to become part of the syncytium by progressive cell wall dissolution during feeding site expansion cannot be recognized. Hence, in this study, we refer to the syncytium and adjacent cell layers as the ‘syncytial area’. The fluorescence signal of ntdTomato was not specific to any type of tissue, as it was observed in the syncytial area as well as in surrounding tissues, i.e., in the epidermis, cortex and vascular bundle outside the syncytial area (Figure 1K). However, a local increase of ntdTomato fluorescence was observed in the syncytial area as compared to the background signal of ntdTomato in normal root tissue.

In contrast to cytokinin, auxin signaling was clearly observed at 4 DPI in the syncytial area but not in surrounding cells (Figure 1J). No auxin signal was observed in the epidermis and cortex, and vascular cylinder outside the syncytial area, indicating a specific local increase in auxin signaling during the onset of feeding site formation. Interestingly, the observed auxin signaling domain seems to overlap with that of the cytokinin signaling domain inside the syncytial area when merging images of the same sample (Figure 1L). No differences were observed between the root tips of infected and uninfected seedlings, both showing a typical expression pattern for auxin and cytokinin, as reported.^46^ Overall, in infected samples, the double-reporter reveals ubiquitous cytokinin signaling throughout the root segment monitored, while auxin signaling is restricted to the syncytial area. When combining these data from individual samples, it seems that high auxin and high cytokinin signaling domains overlap in the syncytial area in contrast to what is observed in root tips.

### Mini-growth chamber design enables continuous live-cell imaging of auxin–cytokinin double-reporter activity in syncytial areas

**Figure 7.**
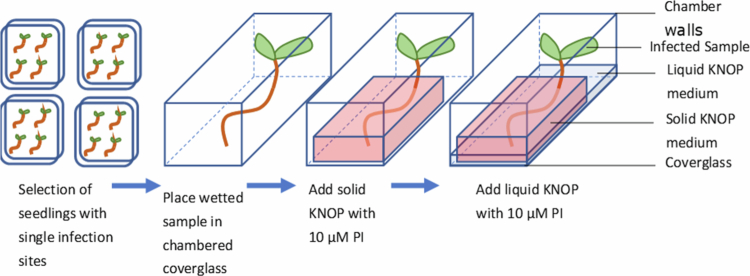
Sample preparation for live-cell confocal imaging. A plant with a single nematode infection that just entered the sedentary stage is selected. It is then stained with propidium iodide, placed in a chambered coverglass, and fixed in place by a block of solid KNOP agar supplemented with 10 μM propidium iodide. The chamber is then filled with liquid KNOP medium with 10 μM propidium iodide to facilitate staining.

Before live-cell imaging of auxin and cytokinin signaling in developing feeding cells began, experimental conditions were established to allow proper growth of uninfected and infected plants, including the support of nematode feeding and development over longer time periods. To enable monitoring of feeding site formation from the onset of nematode parasitism in real-time, *in vitro* root samples were collected with parasitic 2nd stage juveniles that had just ceased migration while their root cells were probed to select an initial syncytial cells. The primary roots of around 200 7-d-old *Arabidopsis* seedlings were inoculated *in vitro,* and after 18 h, the roots were screened using a binocular to select seedlings infected with single 2nd stage parasitic juveniles. Next, individual samples were placed in sterile chambered cover glasses supplemented with both solid and liquid nutrients (Figure 7) to allow further growth and development *in vitro*. Prolonged culturing of plant samples in these mini-growth chambers showed that root growth and the formation of feeding cells continued normally and that juveniles were able to feed, resulting in normal molting into subsequent juvenile life stages. From this, it was concluded that the established method enables normal root parasitism by the cyst nematode *H. schachtii* and could be used for continuous monitoring of the expression of the reporter genes during feeding cell formation. To visualize cell boundaries and facilitate the detection of syncytium formation during imaging, infected roots were incubated with propidium iodide or Calcofluor White. A suitable concentration was determined for each dye by performing a toxicity assay. The inhibition of plant growth was tested by supplementing the growth media with either 10, 50, or 100 μM propidium iodide (PI) or 0.1, 1, or 10% (w/v) Calcofluor White. All concentrations of Calcofluor White and PI at or above 50 μM visibly hampered root growth, as observed after one week of incubation. We concluded that PI is the most suitable cell stain in this setup for *Arabidopsis* plants when it is used at concentrations of 10 μM or below. At 10 μM PI, no visible effects on nematode growth were observed, while still providing proper counterstaining of the plant root cells. Hence, we conclude that the experimental conditions within the mini-growth chamber, as established in this study, allow nematode growth and feeding cell development for prolonged periods of time while facilitating conditions suitable for monitoring gene expression using CLSM (for details, see Materials and methods). To mitigate photobleaching, the microscopic settings were tuned to the cytokinin (stele and columella) and auxin (quiescent center and surrounding cells) signaling maxima in the root tip. The intensity of the laser power was relatively low and set in such a way that these maxima were clearly visible. To minimize the loss of resolution in cells with lower expression levels of the double-reporter, this was compensated for by using the gain settings during image acquisition to make full use of the detector range.

### Live-cell imaging reveals a steep and wide-spread increase in cytokinin signaling upon infection

To study the auxin and cytokinin signaling dynamics during the early stages of feeding site formation, GFP and ndtTomato fluorescence was monitored with an interval of 30 min for a time period of at least 80 h in three independent *H. schachtii* infection sites as well as an uninfected control root segment obtained from the *Arabidopsis* double-reporter line. The resulting timelapse series was compiled into four movies to visualize the spatio-temporal changes in reporter gene expression as an indicator of auxin and cytokinin signaling (Supplementary data). In these movies, the infection process can be observed real-time from the onset of parasitism, including other nematode-induced changes like the asymmetric emergence of multiple secondary roots in close proximity of the syncytial area.^49^ In two samples, the nematodes successfully developed into the fourth juvenile stage and emerged from the root due to an increase in body size, indicating that continuously monitoring with a CLSM laser beam for up to 140 h had no pronounced effects on the feeding process and nematode growth. In one sample, however, the formation of the syncytial area progressed normally until 80 h, after which the host plant died, as inferred by a sudden arrest of nuclear movement and cell permeation by propidium iodide (MovieS1_Rep1). Therefore, data obtained after 80 h were excluded from further analyses for this sample. Additionally, it should be noted that for one replicate, no cell wall counterstaining with PI was used (MovieS3_Rep3).

Over the course of the infection process, the main root thickened, and in two cases, the nematode gradually swelled and developed into a J4, bursting from the root (MovieS2_Rep2 and MovieS3_Rep3). Additionally, in all the samples, 3–6 secondary roots developed in close proximity to each other. Furthermore, a strong increase in ndtTomato fluorescence was noted across the entire sample (Figure 3A–D). The increase in ndtTomato fluorescence was not limited to the stele but was also observed in the cortex and epidermis (Figure 3A–D). This increase is in agreement with the observations made previously from the independent snapshot experiment (Figure 1). During the imaging process, 7–10 optical slices were made every 30 min over a depth of 64 microns, capturing a major part of the syncytium. To compare samples, the head of the nematode was taken as a reference for the starting point of feeding site induction and subsequent development. In this way, a similar part of the syncytial area was compared for all the samples; thus, the optical slice containing the head of the nematode was used as the starting point (Figure 3A–D). In case the head of the nematode gradually changed position from the optical slice during the measurements as a result of sample drift, a switch was made to the desired optical slice. At later stages of infection, the precise position of the nematode's head became invisible by bright-field microscopy because of local root thickening. At these later stages, sample drift was monitored by analyzing the signaling intensities of the entire imaged field between subsequent time points. In the case of unexpected shifts due to sample drift, a switch was made to a neighboring optical slice to restore the gradual trend.

To quantify the observed changes in fluorescence over time in cytokinin signaling, nuclei showing ntdTomato fluorescence signals were converted into the relative mean intensity (RMI_tomato_) and fluorescent area (FA_tomato_). The RMI_tomato_ is the average of the fluorescence intensities of all fluorescent pixels as measured in a single optical slice at a certain time point relative to the same measurement at the first timepoint (Figure 3E). Thus, the RMI_tomato_ reflects the fluorescence intensities in cytokinin signaling as a ratio compared to the first time point (Figure 3E). The FA_tomato_ is defined as the sum of all fluorescent pixels as detected in fluorescent nuclei at a given timepoint for the observed optical slice (Figure 3F). FA_tomato_ reflects the number and size of fluorescent nuclei, which serve as another indicator of cytokinin signaling. Thus, the RMI_tomato_ is a measure of the relative strength of the cytokinin signal within an area compared to the start of the first measurement, whereas the FA_tomato_ is a measure of the total number of cytokinin signaling pixels within an area. The nuclei were selected for pixel measurements by using a mask based on the signaling intensity and a noise threshold cut-off (see Materials and methods). Measuring the cytokinin response in the entire main root in the optical slice with the head of the nematode reveals in all three replicates a steep increase in the RMI during the first 40 h of real-time live-cell imaging (Figure 3E). In this timeframe, the rise in signaling intensities varies between the replicates and ranges between a 2- and 4-fold increase (Figure 3E). After 40 h, the intensity of cytokinin signaling in the nuclei remains relatively stable in two of the three replicates (Figure 3E). For one sample (Rep1), stabilization was already visible after 20 h. This early arrest in cytokinin signaling may be caused by premature starvation of the corresponding plant after 80 h, as described (MovieS1-Rep1). In contrast to the infected samples, the cytokinin signaling (RMI_tomato_) in the control remains constant and is hardly affected during 140 h of live-cell imaging (Figure 3E), indicating that the contribution of stress-related induction of reporter activity due to the growth conditions in the mini-growth chamber can be neglected.

During live-cell imaging, the number of nuclei showing cytokinin signaling increased significantly (Figure 3A–D), most likely due to multiple cell divisions but potentially also by enlargement of existing nuclei caused by endoreduplication.^50^ Both phenomena are reflected in the gradual increase in the total number of fluorescent pixels (FA_tomato_) (Figure 3F). The more than two-fold increase in the FA_tomato_ in the uninfected root can be explained by periclinal and anticlinal cell divisions responsible for secondary growth, as can be viewed by a gradual thickening of the vascular bundle (movieS4_Uninf). In the infected samples, the rise of the FA_tomato_ ranges from 2- to 3-fold. As described in previous studies, syncytial elements are surrounded by cambial and peridermal cells showing extensive proliferation, which may result in a ten-fold increase in cell numbers when compared with neighboring regions outside the syncytial area.^48^ In addition, FA_tomato_ is also influenced by multiple cell divisions, giving rise to the development of secondary root primordia in proximity of the expanding syncytial area (Figure 3C and D, see also movieS3_Rep3). Measuring the signaling area per nucleus demonstrated that the fluorescent area per nucleus also increased during the measurements. Quantification of the fluorescent area per nucleus at the start of live imaging and at 100 h revealed an average increase of 45%, as established by a random selection of 30 nuclei across the infected root at both time points (Supplementary Figure S1).

Overall, our data demonstrate that in both the control as well as in the infected root, the number of fluorescent pixels (FA_tomato_) gradually rise over time while only in the latter, the intensity of the fluorescence (RMI_tomato_) increases. Since ndtTomato fluorescence is an indicator of cytokinin signaling, we conclude that early events of the infection process are associated with a steep increase in cytokinin signaling reaching a plateau, likely reflecting a cytokinin signaling maximum.

### Strong and heterogeneous increase in cytokinin signaling intensity within syncytial area

The next step was to analyze the spatio-temporal dynamics of cytokinin signaling within the syncytial area (Figure 4). To examine the signaling dynamics solely within the syncytial area and exclude the cytokinin signal in the surrounding tissues (e.g., the epidermis, cortex and secondary root primordia), its size and growth were first approximated by manually marking the borders of the syncytial area at four (Rep1 and Rep3) or six (Rep2) timepoints using the bright field channel of the movie. The borders of the syncytial area were subsequently interpolated between these time points. This resulted in a linear approximation of the growth and shape of the syncytial area, thereby providing a simulated shape of the syncytial area for each time point in the movie (Figure 4A–D). The relative intensities of the cytokinin signal (RMI_tomato_) were then measured inside the borders of the syncytial area (Figure 4E). The RMI_tomato_ within the syncytial area shows in all three replicates a pronounced increase, especially during the initial phases of the expanding syncytial area (Figure 4E). The magnitude of the increase varies between the replicates and ranges from 1.5- to about 5-fold, which may reflect the differences in the rate of development of the three individual nematodes and their expanding syncytia. It is noted that the time points at which the first contours of the syncytial area are observed differ among the three replicates (Figure 3E); hence, the start of the RMI_tomato_ measurement within the syncytial areas also varies between replicates (Figure 4E).

In order to gain an indication of the strength of the cytokinin response, the mean signal intensity of the nuclei inside the syncytial area was compared to the mean signal intensity of the nuclei in the root tips of the secondary roots that could be observed within the same sample for all three replicates (Figure 4F). To this end, the mean intensity inside the syncytial area was taken at the point in time at which the highest RMI_tomato_ (Figure 4E) was measured (*t* = 71.5 h (Rep1); 97.5 h (Rep2) and 130.5 h (Rep3)). Then, in the same replicate, the mean intensity of cytokinin signaling was measured in secondary root tips by selecting an optical slice with the highest intensity (see examples in Supplementary Figure S9). As such, the highest measured mean intensity inside the syncytial area was compared to the highest measured mean intensity in the secondary root tips. This comparison revealed that the average cytokinin response at the feeding site is up to five times stronger than that at the root tips of secondary roots.

To study the spatio-temporal dynamics within the syncytial area, the optical slice of the infected primary root sample was divided into 10 optical cross sections (segments) of 90 μm along the length of the syncytial area. Next, the mean fluorescence intensities of the mask-selected nuclei were calculated for each segment only within the boundaries of the syncytial area (Figure 4A–D). The values obtained from the optical slices (that were made every 30 min) were subsequently averaged over 5 h of measurement and plotted as a heatmap (Figure 4G). Noticeably, the mean intensities of the cytokinin response are not homogeneously distributed within the syncytial area, show large variations between segments and may differ more than 7-fold at certain time points (Figure 4G). A similar pattern was observed in the other two replicates (Supplementary Figures S2 and S3). Additionally, within segments, large differences in signaling intensities between individual nuclei are observed (Figure 4A–D), indicating that the syncytial area is composed of a heterogeneous population of (fused) cell types with inherent differences in cytokinin signaling. Additionally, the numerous cell divisions may contribute to the heterogeneity of the signal (‘dilution effect’). It is noted that the microscopic resolution is too low to reveal the identity of the various cell types within the stele and to follow the incorporation of individual cells into the syncytium.

The segments that include the head of the juveniles show in all three replicates (Figure 4G) (Supplementary Figures S2 and S3), which have relatively high signaling intensities. However, high signaling levels are also observed in neighboring segments, which often exceed the signaling level of the segment with the nematode's head. Similarly, the first observed rise of the signal occurs simultaneously in two or more neighboring segments and was not exclusively in the segment with the nematode's head (Figure 4G) (Supplementary Figures S2 and S3). Despite the irregular spatial signaling pattern, nearly all segments show a gradual increase in the cytokinin response over time, with an increase of up to 7-fold (Figure 4G). It is noted that also outside the syncytial area, a strong increase in signaling intensity was also observed in the vascular tissue (Figure 4A–D). In addition, nuclei in the cortex and epidermis also strongly increased (Figure 4A–D).

It can be concluded that the rise in cytokinin signaling is heterogeneously distributed within the syncytial area, and this heterogeneous increase is also observed in surrounding tissues. The signal intensities within the syncytial areas show a steady increase in most segments during the first 70–90 h, after which the signal reaches a maximum level. Altogether, our data indicate that the induction and expansion of a syncytial area is accompanied by a strong cytokinin response in various cell types, including cells that are not incorporated into the syncytial area.

### Auxin signaling starts with a lag phase followed by a moderate and transient rise

Snapshot images of the fluorescent auxin reporter (3nGFP driven by the DR5revV2 promoter) show that GFP fluorescence was only visible in the root tips and secondary root primordia in the uninfected control sample, and that little to no signal was perceived from any other cell types (Figure 1B‒F). A similar situation was seen in the movie of the infected samples at the beginning of the measurement, where no auxin response was detected (Figure 5A). During the course of the experiment, auxin signaling was induced in the infection site as well as in the developing secondary root primordia (Figure 5A–D). It is noted that, apart from secondary root primordia, little auxin signaling is observed outside the syncytial area. To gain insight into the dynamics of this induction, RMI_GFP_ and FA_GFP_ were measured in all three samples using the same procedure as has been followed for quantifying the cytokinin signal. Analysis of the RMI_GFP_ in the optical slice of the main root, which included the head of the nematode (Figure 5E) and emerging secondary root primordia, shows that the auxin response in the three replicates rises to a maximum RMI_GFP_ value between 30 and 70 h depending on the sample and varies between 1.5 and 3. Thereafter, the RMI_GFP_ drops to a slightly lower level in all replicates, which was maintained until the end of the measurements. Notably, during the first hours of the sedentary phase of the juveniles, no auxin signal was detected. Apparently, the observed rise in auxin signaling is preceded by a lag phase of about 20–30 h after the start of the measurements (Figure 5E). As expected, the number of pixels showing auxin signaling (FA_GFP_) rises after the lag phase (Figure 5F) due to an increase in the number of nuclei showing auxin signaling (Figure 1B–F). Similar to cytokinin signaling, the number of pixels showing auxin signaling (FA_GFP_) increases during live-cell imaging in all three infected replicates, although to different levels depending on the sample (Figure 5F). No auxin signal was observed in the uninfected control sample (Figure 5F), showing the specificity of the local auxin response upon nematode infection of the roots.

It is noted that the fluctuations observed in the RMI_GFP_ measurements of the 3rd replicate (Figure 5E) were caused by the additive effect of auxin signaling during the development of secondary root primordia. First, emerging secondary roots contribute to a pronounced rise in auxin signaling when still inside the main root, but a decrease in auxin signaling occurs when the secondary roots grow out from the analyzed area (main root). To exclude the influence of auxin signaling in secondary root primordia, RMI_GFP_ was also measured only within the syncytial area (Figure 5H). Similar to the cytokinin analysis, the syncytial area was manually marked at various time points, and the growth of the syncytial area was interpolated between these marked borders. Next, RMI_GFP_ was determined in the syncytial area, which revealed a pronounced increase in the auxin response reaching maximum RMI_GFP_ values between 2.5 and 4.5 (Figure 5H). After a lag phase, the RMI_GFP_ values of the entire syncytial area reach their maximum around 40 (1st and 2nd replicate) or 70 h (3rd replicate), followed by a decline in auxin signaling to an intermediate steady-state level (Figure 5H).

In order to gain an indication of the relative strength of the auxin response, the mean intensities of the nuclei inside the syncytial area were compared to the mean intensities of the auxin signaling nuclei in the secondary root tips. To this end, the mean intensity inside the syncytial area was taken at the point in time at which the highest RMI_GFP_ was measured (*t* = 42.5 h (Rep1); 46.5 h (Rep2) and 76.5 h (Rep3)) (Figure 5G). Then, the mean intensity was measured within well-developed secondary root tips for comparison (Supplementary Figure S9). Secondary root tips were selected from each replicate, and optical slices with the highest mean intensity were used for comparison. In contrast to the cytokinin analysis (Figure 4F), this comparison reveals that the auxin response within the syncytial area is significantly weaker, up to a factor of five, compared to the auxin response in secondary root tips.

To study the spatio-temporal dynamics of auxin signaling within the syncytial area, the infected root samples were divided into optical cross sections of 90 μm for each time point as described for cytokinin. Although the boundaries of the syncytial area became visible with bright field microscopy at 5 h (2nd replicate), 16 h (1st replicate), and 22 h (3rd replicate) after the start of the measurements, no auxin signal could be detected yet. After this lag phase, the auxin signaling intensities showed an increase in all three replicates and are observed in nearly all segments (Figure 5I). The time points at which the auxin response maxima are reached vary between replicates. Additionally, within a single syncytial area, the auxin signaling intensities are heterogeneously distributed, and the height of the auxin maxima differs between segments. These variations seem to reflect fluctuations in the expansion rate and also the direction of expansion of the individual feeding cells. For example, between 21 and 65 h, the largest expansion in the 3rd replicate was observed in segments 5, 6 and 7 in front of the nematode's head, and later, the syncytial area fully expands in segments 1, 2, 3, and 4 below the main body of the nematode (Figure 5B–D). This differential expansion is in part also reflected in the heatmap of the segment analysis (Figure 5I). It is noted that this pattern is also observed for the cytokinin signal (Figure 4G). No evidence is obtained for nematode hormone secretion, as no consistently stronger fluorescence signal is observed around the head of the nematode compared to other areas. In all three replicates, elevated levels of auxin signaling are observed in the segment containing the head of the nematode (Supplementary Figures S4 and S5). However, high auxin signal intensities are also observed in two or more neighboring segments, often exceeding the segment harboring the nematode's head. In addition, the first increase in auxin signaling intensities does not originate from a single segment and is also observed in two or more, often neighboring, segments. Additionally, the decline in auxin signaling occurs nearly synchronously in neighboring segments.

Overall, it can be concluded that the transient rise in auxin signaling is restricted to the syncytial area and is preceded by a lag phase during which the first contours of the syncytial area become visible with bright-field microscopy. From this, we infer that the initial phase of syncytia development is independent of auxin signaling or accompanied by very low auxin signaling levels below the GFP detection threshold of the CLSM used. In contrast to those in the cytokinin signaling, the auxin signaling intensities are relatively low in the syncytial area. A comparative analysis showed that the auxin signaling intensities were significantly lower in the syncytial area than in the secondary root tips, while for the cytokinin response, the opposite was observed.

### Rise in cytokinin signaling within syncytial area precedes an increase in auxin signaling

To understand the differences in cytokinin and auxin signaling in more detail, the nuclei were divided into three types that showed either auxin signaling, cytokinin signaling, or both (Figure 6A–D). Next, the number of pixels (FA) displaying auxin signaling, cytokinin signaling or both was quantified in the entire imaged main root. At the end of the migratory phase, at the start of the measurements, all the nuclei show only a cytokinin response (Figure 6E). Moreover, during the steep increase in cytokinin signaling, no auxin signaling nuclei are observed in the primary root. After 30 h a rise in the number of nuclei displaying both auxin and cytokinin responses are observed (Figure 6E). Only cells that show an auxin response but no cytokinin signal are observed in developing secondary root primordia (Figure 6E and Supplementary Figure S6). It is noted that the observed decrease in the FA_GFP_ values of these auxin signaling nuclei after 70 h (Figure 6E) is due to the outgrowth of the secondary roots (Figure 6C and D), and as a consequence, their root tips showing auxin signaling, moved outside the analyzed microscopic field.

To compare the differences in auxin and cytokinin signaling dynamics, FA_GFP_ (Figure 5F) and FA_tomato_ (Figure 3F) were combined in a single figure (Figure 6F). Integration of data for each time point was possible given the simultaneous monitoring of both auxin and cytokinin signaling in the same infected root using a double-reporter line. This comparison confirms that the relative increase in cytokinin signaling starts earlier than the increase in the auxin response. The cytokinin response is continuously rising from the start of the first measurements (*t* = 0) in all three replicates, while the auxin signal is observed later. For example, upon the first appearance of the syncytial area (*t* = 21.5 h; Rep3), the level of cytokinin signaling is already high, with a fluorescent area near 15,000 pixels, while the auxin signal is still absent. The rise in auxin signaling starts at 30 h in the 1st replicate, 25 h in the 2nd replicate, and 35 h in the 3rd replicate, after which the auxin response increases in all three replicates (Figure 6F and Supplementary Figure S7). These findings indicate that the cytokinin signal precedes the auxin signal after the first contours of the syncytial area become visible with bright field microscopy.

In order to determine the correlation between auxin and cytokinin signaling during infection, the mean intensity (MI) of both auxin and cytokinin signaling was determined in the main root for each timepoint (Figure 6G, Supplementary Figure S8A and B). It was noticed that high auxin signaling is associated with high cytokinin responses. However, the inverse is not always true, as high cytokinin intensities do not necessarily always correlate with high auxin signaling intensities. For example, cortical and epidermal cells with a high cytokinin response do not show auxin signaling. Because auxin signaling is contained in the syncytial area, which mainly reflects the relationship between auxin and cytokinin signaling within the syncytial area. As such, this suggests that the induction of auxin signaling co-occurs with an increase in cytokinin signaling within the syncytial area. Moreover, our imaging data (Figure 6A–D) indicate that within the syncytial area, auxin and cytokinin signaling occurs simultaneously in the same nuclei. To further elucidate the ratio between auxin and cytokinin signaling, their mean intensities were measured in individual nuclei (Figure 6H). Nuclei with high auxin and high cytokinin signaling intensities within the syncytial area were selected, and the mean fluorescence intensities within these nuclei were compared to those of nuclei with high auxin or cytokinin signaling in secondary root tips within the same replicate (Supplementary Figure S9A and B). The selection of high auxin or high cytokinin signaling nuclei within the syncytial area and secondary root tips was done by selecting the nuclei having the majority of pixels with signaling intensities representing the highest 10% of the measured intensities. The selection of nuclei within the syncytial area was based on using cytokinin signaling intensities (Supplementary Figures S9A and C) as well as auxin signaling intensities (Supplementary Figure S9B and D). The intensities were measured at relatively late timepoints, after the major increase in signaling intensities within the syncytial area had occurred. From the comparison between the different groups of nuclei it can be concluded that within the syncytial area, high auxin signaling values are associated with a high cytokinin response and vice versa, in contrast to what is seen in the root tips (Figure 6H, Supplementary Figures S10A and B). In root tips, nuclei in the root cap and developing vascular tissue with a high level of cytokinin signaling are accompanied with relatively low levels of auxin signaling, while the reverse is the case for nuclei in the quiescent center and neighboring cells with a high auxin response. Overall, it can be concluded that selection of nuclei within the syncytial area with high auxin or high cytokinin signals results in cells with similar auxin‒cytokinin ratios, which are distinct from the auxin‒cytokinin ratios observed in the signaling maxima of the root tips (Figure 6H, Supplementary Figures S10A and B). More precisely, although the auxin response within the syncytial area is relatively low compared to root tips, the highest auxin signaling intensities are not accompanied by a decrease in cytokinin signaling, as observed in the root tips. The reverse applies to cytokine signaling: high cytokinin signaling within the syncytial area is not linked with low auxin responses. It is noted that this pattern is less pronounced in the 1st replicate (Supplementary Figure S10A) than in the other two replicates (Figure 6H and Supplementary Figure S10B), which might be caused by a less vital syncytium as the host plant died after 80 h.

## Discussion

Here, we developed and tested a novel live-cell imaging method to study simultaneously the spatio-temporal dynamics of auxin and cytokinin signaling in plant roots during the initiation of syncytia induced by the cyst nematode *H. schachtii*. Upon infection of a transgenic *A. thaliana* line harboring a nuclear-targeted dual reporter, the auxin and cytokinin signaling responses could be monitored continuously in a single specimen by confocal laser scanning microscopy (CLSM), and images were obtained at 30-min intervals. Quantitative analyses of the optical slices revealed an early wide-spread increase of the cytokinin response during infection, while auxin signaling showed a lag phase and was restricted to the syncytial area. Auxin signaling is observed after the first contours of the syncytial area become visible and reaches its maximum within 70 h and subsequently decreases to a relatively constant level. In contrast, the cytokinin signaling intensities rise early during infection and stay at a constant level afterwards. Interestingly, a comparative analysis shows that the number of cytokinin signals in the syncytial area are higher than the number of cytokinin signaling maxima in secondary root tips, while for auxin the response is lower. In addition, we show that within the syncytial area, auxin signaling maxima are paired with high cytokinin signaling intensities, which contrasts with the auxin and cytokinin ratios seen in the root tip. Altogether our data indicate that auxin signaling increases after the first contours of the syncytial area become visible, suggesting that auxin plays a role during expansion of the syncytium rather than initiation. The widespread occurrence of cytokinin signaling, also outside the vascular tissue, may point at multiple roles during infection, which may include initiating and enlarging the syncytial area as well as regulating early defense responses. Overall, our data suggest that cytokinin signaling plays a role in syncytium formation before auxin does. This necessitates a shift in our current view of the role of cytokinin and auxin in syncytium induction and development, as previous studies have suggested that the initial steps of syncytium formation are dependent on auxin.^13^^,^^16^ Instead, our data seem to support a model in which rapid and widespread cytokinin signaling in infected root segments may reflect the preconditioning of root cells that are incorporated into the syncytium, which are known to undergo rounds of cell division early after infection.^11^ Although cytokinins are often described as inhibitors of cell division in roots, they can also promote cell division in certain root tissues like in shoots.^51^ Thus, the observed induction of auxin signaling confined to the syncytial area may reflect its contribution to cell expansion rather than to cell division once preconditioned cells are incorporated into the growing syncytium.^52^

In this study, an increase in cytokinin signaling was observed throughout the entire feeding site within the first day of measurement (Figure 3E). These findings are in accordance with prior studies employing specific reporter genes, such as TCSn:GFP and ARR5:GUS, which have demonstrated an upregulation of cytokinin signaling within the syncytium of *H. schachtii* in *A. thaliana* as early as 2 and 3 d post inoculation (dpi), respectively.^26^^,^^27^ This is roughly the same timeframe used in our study, as we introduced the nematodes into the sample approximately 18 h prior to live-cell imaging (*t* = 0). Thus, a day after the start of live-cell imaging corresponds to a time point of around two days post inoculation. With regard to the spatial distribution of cytokinin signaling, we observed a fluorescent signal in the cortical and epidermal cells during live-cell imaging (Figure 3A–D). In contrast, previous reports on cyst nematodes did not show the presence of cytokinin signaling in cortical and epidermal cells despite the use of the same nematode and plant species.^24^^,^^26^ However, the key difference might be the use of different promotors or fluorophores in the reporter gene constructs. In the case of the ARR5:GUS reporter, the TCSn promotor was shown to be more sensitive to cytokinin signaling compared to several ARR promotors.^41^ Furthermore, the ntdTomato fluorophore is up to six times brighter than EGFP and, as such, it is likely that we could visualize responses to lower cytokinin concentrations than the use of TCSn:GFP.^53^ Alternatively, the difference might be explained by the fact that the TCSn promoter also responds to plant stress, which results in expression in the epidermis and cortex. The epidermal and cortical cytokinin signal might thus be explained by an abiotic stress response induced by the measurement setup, as this is seen in both the infected as well as the uninfected samples. A possible source of stress within the measurement setup could be regular exposure to laser light in addition to growth under *in vitro* conditions. Additionally, light might affect root development mediated by cytokinin via the expression of the cytokinin independent-1 (CKI1), which in turn is regulated by light through PhysA.^54^

Cytokinin signaling induction during parasitism is not exclusive to *H. schachtii*, and is also observed during the formation of giant cells induced by root-knot nematodes.^24^ Moreover, an early and rapid induction of cytokinin signaling is also seen in other biotic interactions. For example, early activation of cytokinin signaling is observed in plants invaded by parasitic plants like *Phelipanche ramose*, where cytokinins play a pivotal role in prehaustorium induction.^55^ Cytokinin levels and signaling are also induced upon wounding, leading to a more widespread cytokinin response during herbivory by larvae of the moth *Manduca sexta.*^56^ In the case of *Lotus japonicus*, the TCSn:YFPnls cytokinin reporter showed a progressive fluorescent signal in response to *Rhizobium* bacterial colonization from 2 dpi onwards. The signal was located in foci of dividing cortical cells, which correspond to the development of nodule primordia.^57^ Previously, it has been suggested that the induction of cytokinin signaling during cyst nematode infection contributes to both developmental processes and defense.^26^ Thus, the wide-spread cytokinin accumulation within and around syncytia, as observed in our study, might reflect a dual role for cytokinin in the syncytial development and defense responses induced by the invading nematode. However, the use of the auxin/cytokinin double-reporter in this study does not allow us to untangle defense and developmental responses upon nematode root infection, as they are expected to partially overlap in space and time.

Our data show that the cytokinin signaling intensity decreases when further removed from the nematode's head and nearing the edges of the syncytial area (Figure 4G). As such, the decrease near the edge of the syncytial area might be the result of the injection of cytokinin into the feeding site, as this pattern is in line with what could be expected upon the release of cytokinin through the nematode's stylet into the initial syncytial cells. For *H. schachtii*, the biosynthesis and release of cytokinin was reported, probably to control feeding site formation.^27^ Cytokinins have been measured in *H. schachtii*, and a cytokinin-synthesizing isopentenyltransferase gene (HsIPT) has been identified. Silencing of this gene lowers the level of free base *iP*, an active form of cytokinin, within juveniles and attenuates the infection process, which is consistent with a possible role for cytokinin secretions in the induction of cytokinin signaling during the onset of parasitism, as observed in our study. Alternatively, local plant-based biosynthesis of host cytokinins might explain the distribution of the observed cytokinin signal. Infection by *H. schachtii* was shown to upregulate cytokinin biosynthesis (IPT) gene expression in *A. thaliana.*^24^ As such, the increased cytokinin signaling near the nematode’s head might be the result of increased local cytokinin biosynthesis or the release of cytokinin by the nematode, or even a combination of the two.

Similar to cytokinin, auxin signaling dynamics could be studied in time and space using the double-reporter. The live-cell imaging data shows the induction of auxin signaling within the syncytium of *H. schachtii* during infection at 2 to 3 dpi (Figure 5E). However, auxin accumulations have been reported as early as 18 h post inoculation while our live-cell data show accumulation between 2 and 3 dpi.^16^ This difference might be explained by variations in the migration process, as this study included only parasitic juveniles near the end of the migratory phase at 18 hpi while nematodes in other studies might not have experienced this delay and may have started earlier with the induction of syncytia. Alternatively, early auxin induction^16^ might perhaps be attributed to the GUS reporter, as the expression of this enzyme results in a signal amplification due to the conversion of the substrate into a stable product, in contrast to the more gradual accumulation of a fluorophore, which may need more time to reach the threshold for detection. In addition to plant‒nematode interactions, the induction of auxin signaling has also been observed in other biological systems. For instance, an initiation of auxin signaling was observed during nodule formation by Rhizobium.^58^ Similarly, in the case of the parasitic plant *Striga hermonthica*, the induction of auxin signaling is a fundamental aspect for the switch of *S. hermonthica* roots to form invasive prehaustoria.^59^ Together, these examples, including our findings, underscore the role of auxin signaling in the establishment of symbiotic and parasitic interactions in plant roots by contributing to the development of specialized structures required for nutrient exchange.

Comparable to cytokinin, although later in time, a relatively strong auxin signal is observed in segments close to the head of the nematode (Figure 5I). This might be due to the potential injection of auxin by the nematode into the plant tissue, as suggested by the report on auxin in stylet secretions in previous studies.^60^ Alternatively, auxin accumulations might be the result of plant-based processes including the re-localization of PIN proteins, which act in polar auxin transport. The role of auxin export proteins during nematode infection has been well-documented.^20^ For example, during cyst nematode infection, it is thought that PIN1 is responsible for the flow of auxin into the syncytium, while PIN3 and PIN4 may distribute auxin throughout the feeding site. However, the accumulation of auxin can also be the result of both nematode- and plant-based activities. For example, auxin transport is affected by the nematode effector 19C07 from *H. schachtii*, which was reported to interact with the auxin influx transporter LAX3.^61^ Finally, increased local auxin biosynthesis might also play a role. For example, the upregulation of the IAM and YUCCA auxin biosynthesis genes is observed during infection of *H. schachtii* and *M. incognita.*^22^ Additionally, ethylene response factor 9 (ERF109) promotes auxin biosynthesis by binding to the promoters of YUC2 and ASA1 and has been suggested to regulate local auxin biosynthesis at the nematode infection site.^49^ This suggests that the auxin accumulation in syncytia is at least partially the result of local auxin biosynthesis. As such, the relatively strong auxin signal in segments near the head of the nematode as observed in this study might be the result of hormone injection, increased local biosynthesis or transport manipulation.

Aside from the analysis of the signaling dynamics of individual hormones, the use of the double-reporter has allowed us to study auxin and cytokinin signaling simultaneously in a single nematode infection site. Using this reporter, we observed an increase in auxin signaling during infection, but its intensity was significantly weaker compared to that in secondary root tips (Figure 5G). In contrast, cytokinin signaling inside the syncytial area seemed to exceed the level detected inside the root tips (Figure 4F). This might be an indication that cytokinin signaling is more prevalent during nematode infection compared to auxin. The differences in auxin and cytokinin signaling in the syncytial area seem to be consistent with earlier data based on the transcriptional analysis of nematode feeding sites. Comparative analysis of feeding sites induced by root-knot nematodes and feeding sites induced by cyst nematodes revealed that cytokinin related genes are enriched compared to auxin related genes. For example, LBD16 and its positively co-regulated genes were repressed in syncytia while in contrast cytokinin-induced genes were enriched.^18^ This supports our data that cytokinin plays a more dominant role in syncytium development. Interestingly, for gall formation by the root-knot nematode, the opposite was shown, indicating that auxin might be a more dominant factor in giant cell formation.^18^ While the strength, tissue and timing of the hormone signals seem to differ in infected root segments, a substantial part of the auxin and cytokinin signaling domains overlap in time and space. We could even demonstrate that auxin and cytokinin signaling occur simultaneously in single nuclei located inside the syncytial area during the onset of feeding site development (Figure 6A–D). Interestingly, nearly all of the auxin signaling that is induced due to nematode infection seems to be co-localized with cytokinin signaling at the cellular level (Figure 6E). The absence of nuclei inside the syncytium, in which only auxin signaling occurs and the auxin signal is always paired with a high cytokinin signal on an individual cell level, suggests that the role of auxin during nematode development is through interplay with cytokinin. Previous reports have shown that in developing vascular tissues of root tips, auxin and cytokinin signaling maxima do not overlap, in contrast to what is observed within the syncytial area.^62-64^ In the developing vascular tissue of the root tip, auxin signaling is confined to metaxylem and protoxylem cells, while neighboring procambium cells and phloem cells display cytokinin signaling.^62^^,^^63^ Similar patterns are observed in the root cap, quiescent center and neighboring stem cells, though the precise position of the signaling maxima may vary depending on the type of reporter.^41^^,^^42^^,^^47^^,^^65-67^ For example, the DR5 reporter has the highest response in the quiescence center, while the DR5revV2 reporter has its maxima in subtending columella cells.^47^ Additionally, direct measurements and predictive modeling of auxin and cytokinin concentrations may lead to deviations from reporter gene expression studies.^68^ A well-known example is the quiescence center where high cytokinin concentrations have been measured, while the cytokinin response is relatively low, and is presumably repressed by high auxin concentrations via AHP6 signaling.^68^ Nevertheless, the expression profiles of reporters are widely accepted to study the interaction between auxin and cytokinin but should be interpreted in the appropriate context and in a comparative way without referring to actual hormone concentrations. Therefore, it is feasible to conclude that the overlapping auxin and cytokinin signaling maxima revealed by the double-reporter indicate that, in contrast to those in the root tips, the signaling modules leading to the separation of these maxima are not operating within the syncytial area. Altogether, the pairing of auxin signaling maxima with high cytokinin responses observed in this study highlights the unique features of feeding cell development.

Despite their divergent functions, the interplay between auxin and cytokinin is known to be responsible for developmental processes such as lateral root formation, vascular patterning, and shoot and root meristem development.^35^^,^^62^^,^^69^ As such, not the individual actions of auxin and cytokinin but the interplay between them might explain various processes that occur during feeding site formation like the formation of phloem. It has been speculated that the difference in the ratio of auxin and cytokinin between galls and syncytia affects the formation of phloem but not the feeding site itself.^28^ The phloem is induced around syncytia and giant cells to support feeding site and nematode development. Giant cells are symplastically isolated and obtain nutrients through transporter-mediated processes while, in contrast, syncytia are connected to the phloem by plasmodesmata. It was shown that the phloem around giant cells does not respond to cytokinin while the phloem around syncytia does.^28^ As such, the process of vascularization around feeding sites for cyst and root-knot nematodes could potentially arise from the fine-tuned interplay between auxin and cytokinin signaling pathways.

Finally, as shown in this study the use of an auxin‒cytokinin double-reporter represents a potent method for investigating hormonal signaling dynamics in plant–nematode interactions. However, it is vital to acknowledge and address certain inherent pitfalls in this approach and how these were overcome to ensure the precision and reliability of the acquired data. One primary concern in employing the auxin–cytokinin double-reporter system is the potential impact of laser-induced photobleaching. Photobleaching, the loss of fluorescence due to laser exposure, can compromise the accuracy of signal quantification. To mitigate this, in this study, careful laser power adjustments were made to minimize photobleaching and thereby preserving the integrity of the fluorescent reporters. Even with adjusted laser power, allowing the host plant to grow and develop while being imaged is essential for live-cell imaging. To this end, a chambered coverglass was used in combination with growth medium and agar. This allowed the plants to grow under sterile conditions without the need for external interference. Tissue depth-related variations in the fluorescent signal are mitigated in this study because of controlled comparisons within the same optical layer. This eliminates the influence of tissue depth on signal intensity, ensuring reliable data from the same area. Depth-related variations as a result of sample drift are corrected for by imaging a stack of optical slices around the initial depth of interest. This ensures that corrections for z-drift become possible in the later analysis. The use of DR5revV2:n3GFP-nls and TCSn:ntdTomato-nls double has allowed us to image auxin and cytokinin signaling concurrently. This approach has not been used previously to study hormone signaling in plant–nematode interactions. Hence, we were able to determine on the cellular level that auxin signaling correlates with a high level of cytokinin signaling, which would otherwise have stayed unnoticed. So, in conclusion, the auxin‒cytokinin double-reporter system combined with live-cell imaging is a valuable tool, as shown in this study, for obtaining novel insights into the spatio-temporal dynamics of hormone signaling domains in the context of nematode parasitism. In addition, our analyses show that even an intractable biological system, such as feeding cell induction – characterized by non-uniformity and unpredictable expansion rates and directions – is amenable for quantitative live-cell imaging. This shows that our approach also offers opportunities to study other complex plant‒microbe interactions with capricious developmental patterns at the cellular level.

## Supplementary Material

Supplementary material Supplemental_Movies.zip

Supplementary material Supplementary_Figures.pdf
