# Supplementary material for: Quantitative live-cell imaging of auxin and cytokinin signalling in developing feeding sites of Heterodera schachtii
Source: Plant Signal Behav. 2026 Feb 23;21(1):2629039. doi: 10.1080/15592324.2026.2629039 (PMC12931903; doi:10.1080/15592324.2026.2629039)
Supplement: Supplementary material — Supplementary_Figures.pdf [file KPSB_A_2629039_SM3662.pdf]

Supplementary Data

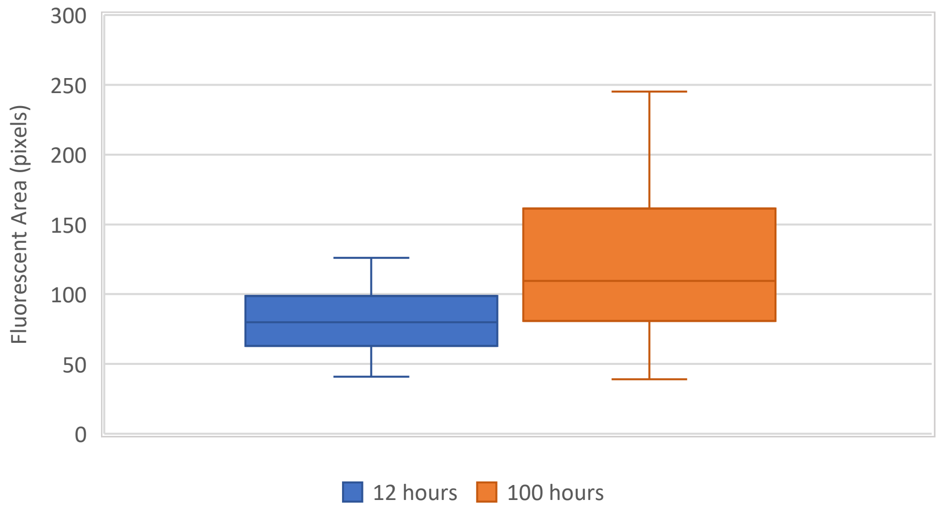

Figure S1 Sizes of the fluorescent nuclei for the 3rd replicate at 12 and 100 hours. Measurement is based on the signal from the TCSn::ntdTomato-nls reporter for cytokinin of thirty nuclei per timepoint.

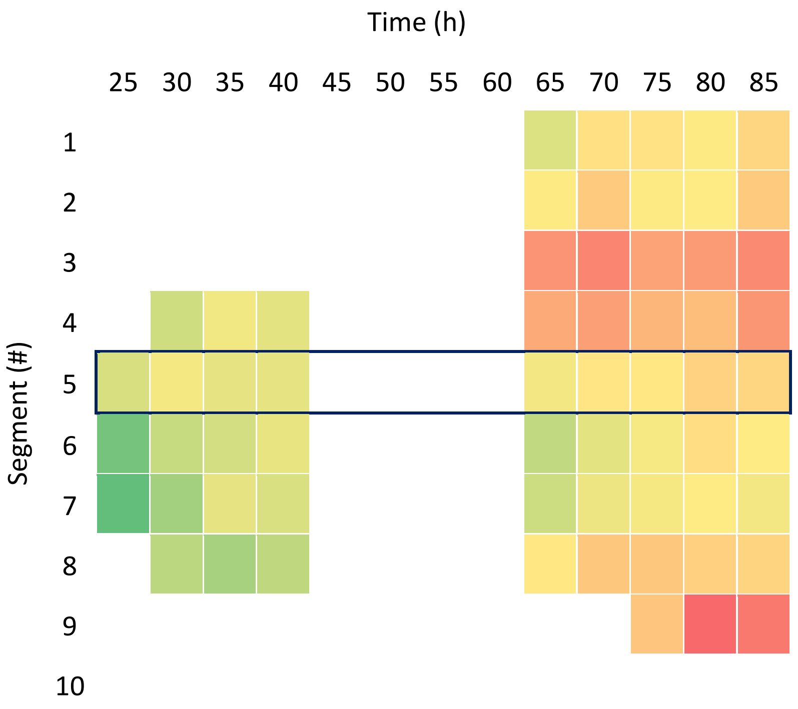

Figure S2 Heatmap of the average fluorescent intensity of the cytokinin reporter within the sy[illegible] over time for the 1st replicate. Severe sample drift that could not be corrected makes the 45-[illegible] therefore not shown. Red indicates a higher intensity and segments are 90 µM long. The segm[illegible] nematode is highlighted in blue.

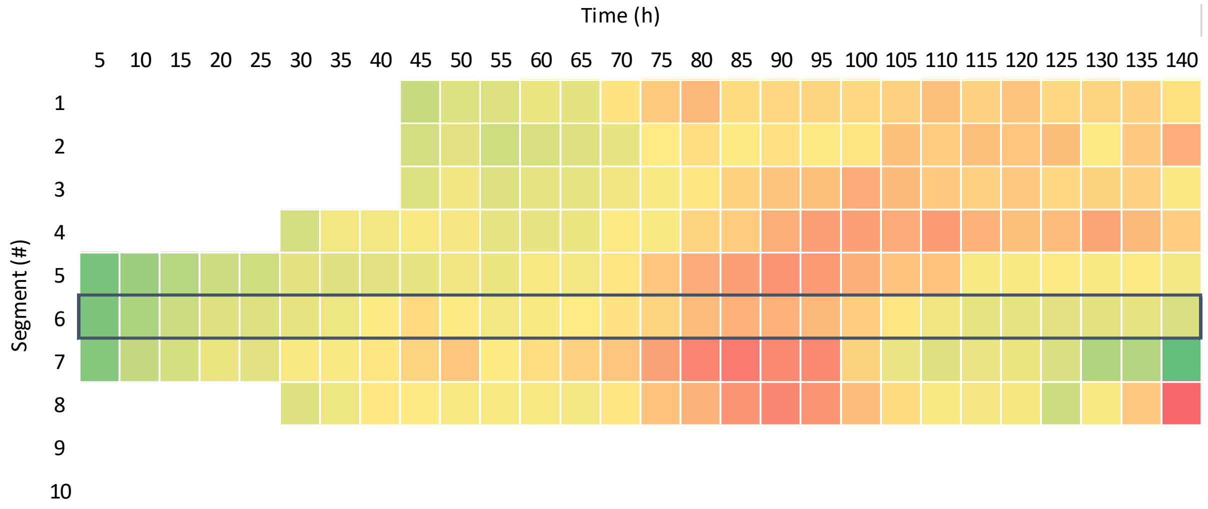

Figure S3 Heatmap of the average fluorescent intensity of the cytokinin reporter within the syncytium per root segment over time for the 2nd replicate. Red indicates a higher intensity and segments are 90 µM long. The segment with the head of the nematode is highlighted in blue.

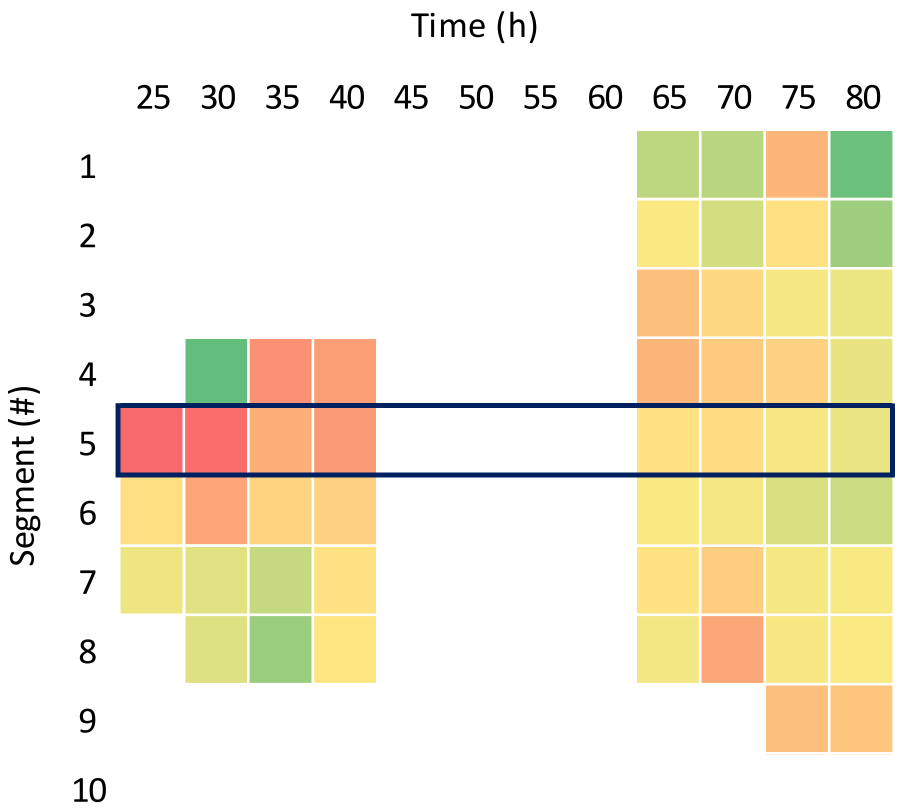

Figure S4 Heatmap of the average fluorescent intensity of the auxin reporter within the syncytium per root segment over time for the 1st replicate. Severe sample drift that could not be corrected makes the 45-60 range unreliable and is therefore not shown. Red indicates a higher intensity and segments are 90 µM long. The segment with the head of the nematode is highlighted in blue.

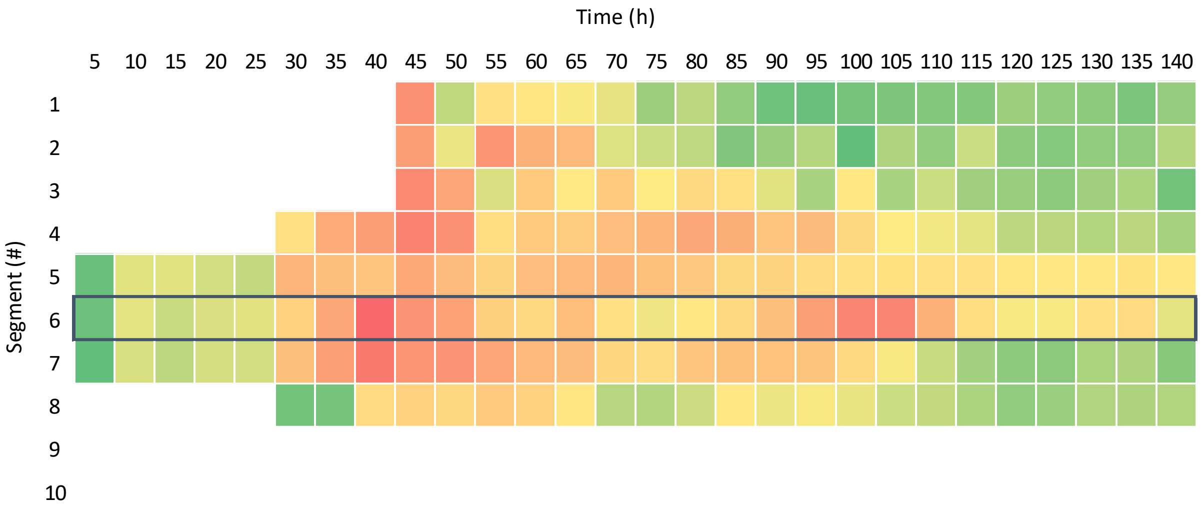

Figure S5 Heatmap of the average fluorescent intensity of the auxin reporter within the syncytium per root segment over time for the 2nd replicate. Red indicates a higher intensity and segments are 90 µM long. The segment with the head of the nematode is highlighted in blue.

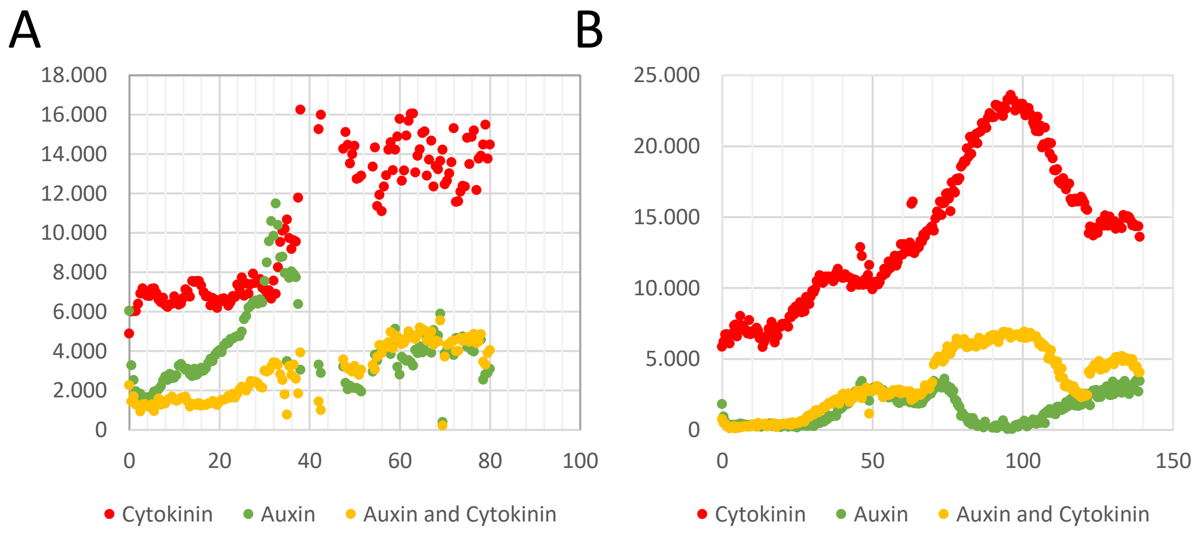

Figure S6 Fluorescent area (FA) as an indication of the number of nuclei that show auxin signalling, cytokinin signalling or both as measured in the main root over time for the 1st replicate (**A**) and the 2nd replicate (**B**). The rise in green FA signal being attributed to the developing secondary root primordia

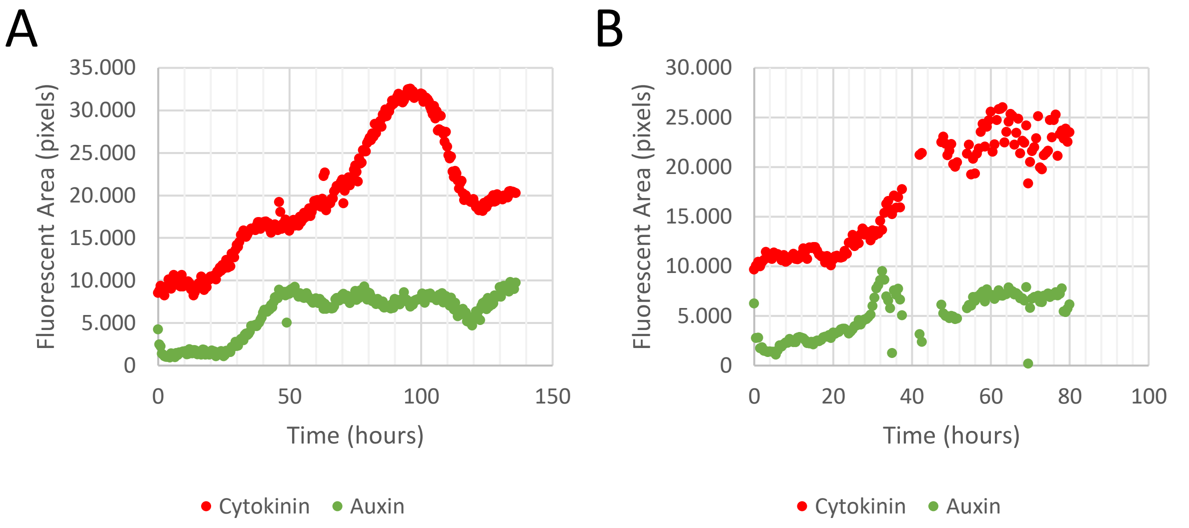

Figure S7 Fluorescent area (FA) of auxin and cytokinin in the main root showing that auxin signalling comes up later than cytokinin in the first replicate (**A**) and the second (**B**).

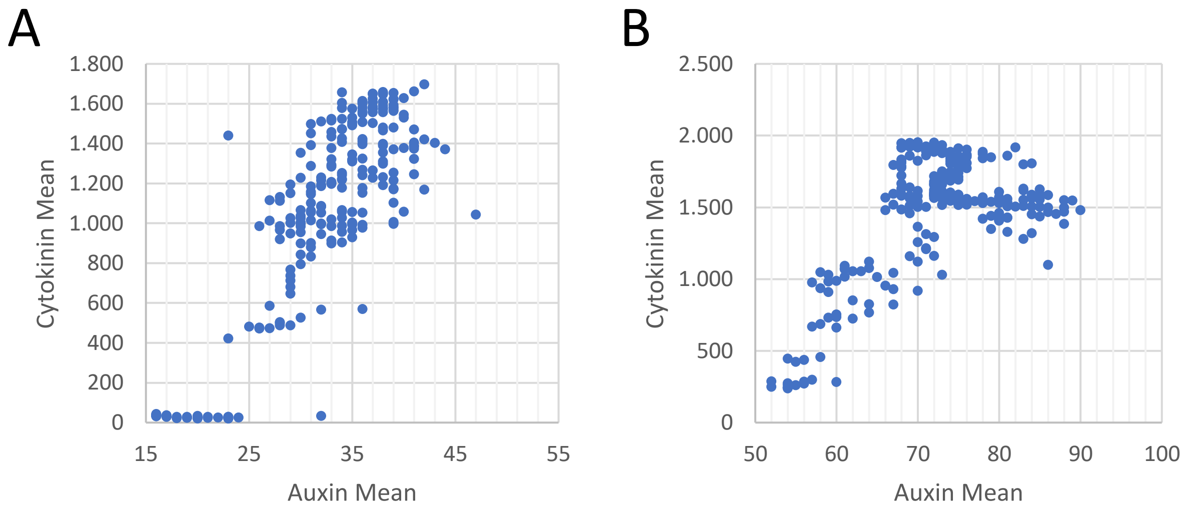

Figure S8 The mean intensity of the cytokinin signal plotted against the mean intensity of the auxin signal in the entire main root for all measured time points of the 1st replicate (**A**) and the 2nd replicate (**B**). This shows the auxin and cytokinin ratios measured at intervals of 30 min during a period of 140h.

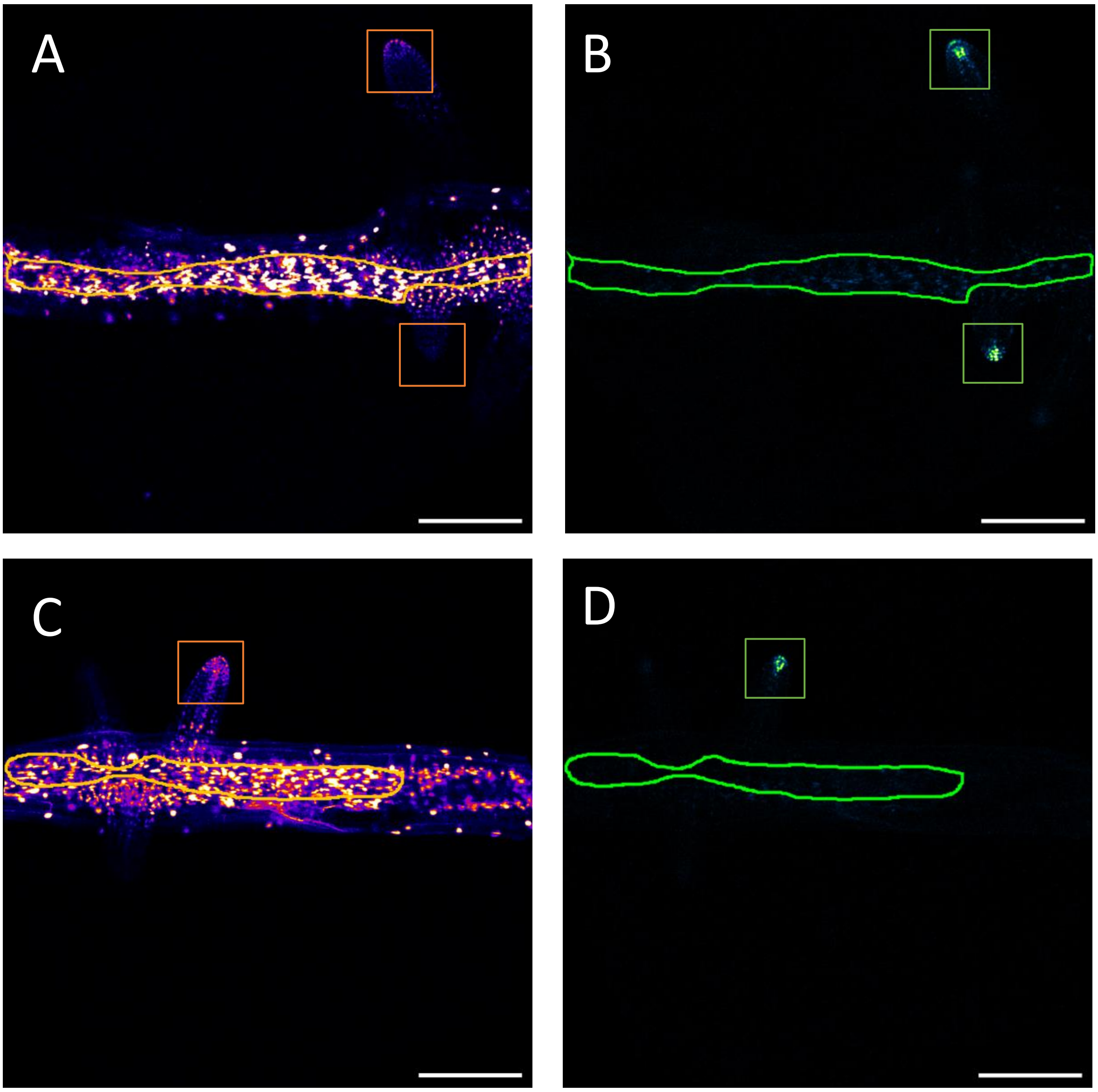

Figure S9 Examples of still frames of two movies wherein the syncytial areas and areas of secondary root tips are indicated in which the fluorescence was measured to compare the auxin and cytokinin signalling intensities between feeding sites and secondary root tips (see Figure 5G, Figure 6H). **S9A** and **S9B** show nuclei with cytokinin (**A**) and auxin (**B**) signalling of the 3rd replicate (t=113h). **S9C** and **S9D** show nuclei with cytokinin (**C**) and auxin (**D**) signalling of the 2nd replicate (t=83h). Cytokinin signalling is displayed as a blue<orange<white (fire) gradient, while for auxin signalling a is displayed as a blue<green<white (green fire blue) gradient. The scale bar represents 200µM.

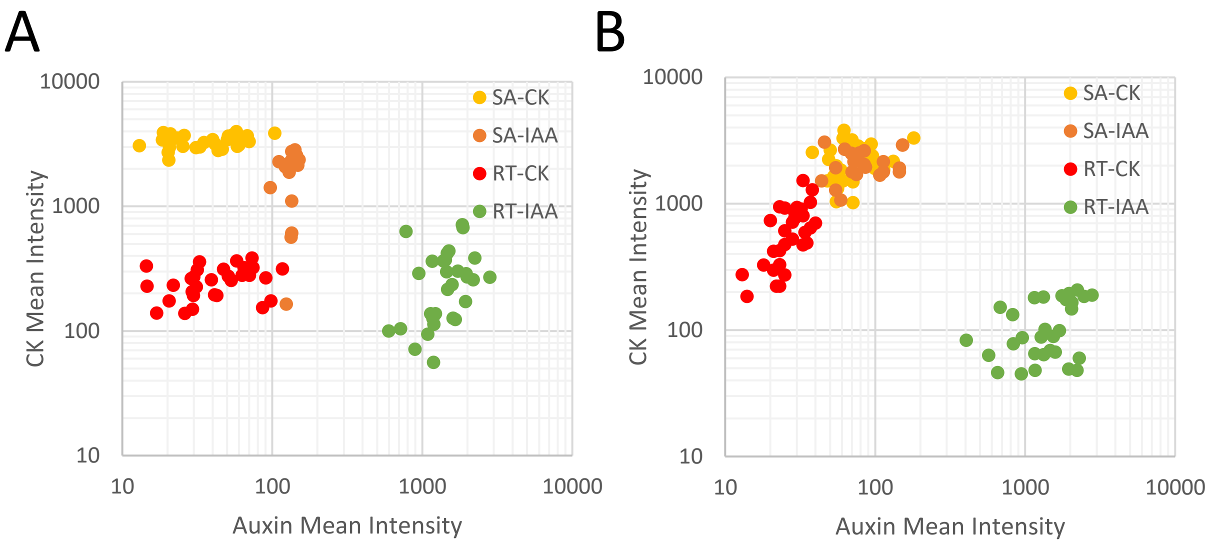

Figure S10 The mean intensity of auxin and cytokinin signals found at a single time point in single nuclei of the 1st replicate at 37,5 hours (**A**) and the 2nd replicate at 31,5 hours (**B**). At least thirty nuclei were selected from the top 10% highest auxin and cytokinin signalling nuclei located in the syncytial area (NFS-IAA and NFS-CK) as well as nuclei present in secondary root tips (RT-IAA and RT-CK) resulting in three groups of cell types based on their auxin-cytokinin ratios. Further explanation see main text. Abbreviations: IAA – Indole-3-acetic acid, CK – Cytokinin, SA – Syncytial Area, RT – Root Tip
